# Design of Conjugates Based on Sesquiterpene Lactones with Polyalkoxybenzenes by “Click” Chemistry to Create Potential Anticancer Agents

**DOI:** 10.3390/molecules27238411

**Published:** 2022-12-01

**Authors:** Margarita E. Neganova, Ekaterina V. Smirnova, Elena V. Sharova, Oleg I. Artyushin, Yulia R. Aleksandrova, Ekaterina Yu. Yandulova, Natalia S. Nikolaeva, Valery K. Brel

**Affiliations:** 1 Nesmeyanov Institute of Organoelement Compounds, Russian Academy of Sciences, 119991 Moscow, Russia; 2 Institute of Physiologically Active Compounds at Federal Research Center of Problems of Chemical Physics and Medicinal Chemistry, Russian Academy of Sciences, 142432 Chernogolovka, Russia

**Keywords:** sesquiterpene lactones, dehydrocostuslactone, alantolactone, azides, acetylenes, click chemistry, cytotoxicity, mitochondria, glycolysis

## Abstract

Using the methodology of “click” chemistry, a singular method has been developed for the synthesis of unique conjugates based on sesquiterpene lactones: dehydrocostuslactone and alantolactone with polyalkoxybenzenes. To expand the structural range of the resulting conjugates, the length of the 1,2,3-triazole spacer was varied. For all synthesized compounds, the cytotoxic profile was determined on the cell lines of tumor origin (SH-SY5Y, HeLa, Hep-2, A549) and normal Hek 293 cells. It was found that the compounds based on alantolactone **7a**–**d** with a long spacer and substances containing dehydrocostuslactone **10a**–**d** with a short spacer have the greatest toxic effect. The decrease in cell survival under the action of these conjugates may be due to their ability to cause dissipation of the transmembrane potential of mitochondria and inhibit the process of glycolysis, leading to cell death. The obtained results confirm the assumption that the development of conjugates based on sesquiterpene lactones and polyalkoxybenzenes can be considered as a promising strategy for the search for potential antitumor agents.

## 1. Introduction

Products of natural origin have always aroused the interest of researchers as sources of biologically active substrates with a wide pharmacological profile. One of the most striking examples of the successful use of natural compounds as effective therapeutic agents is paclitaxel (Taxol), an alkaloid isolated from the bark of the Pacific yew. Anticancer drugs based on taxol are currently widely used in clinical practice [1,2]. In the treatment of malaria, a drug based on artemisinin, a compound belonging to a promising class of natural compounds, sesquiterpene lactones, has given a good account of itself [3,4]. Professor Tu Youyou was awarded the Nobel Prize in Physiology or Medicine in 2015 for the discovery of artemisinin and the study of its semi-synthetic derivatives.

Some sesquiterpene lactones and their derivatives also have antitumor properties; for example, a bioavailable form of parthenolide (dimethylaminoparthenolide) [5], thapsigargin [6], as well as an antitumor drug based on the sesquiterpene γ-lactone arglabin [7]. It has been shown that the biological effect of these sesquiterpene lactones is realized through the induction of apoptosis as a result of their influence on the formation of reactive oxygen species, which leads to oxidative damage in the cell and triggering the mitochondria-dependent pathway of apoptosis [8].

All these examples of the successful application of sesquiterpene lactones as potential antitumor drugs arouse great interest in compounds of similar structure and in their structural modifications [9,10,11,12,13]. Since most sesquiterpene lactones contain an activated *exo*-methylene moiety, which readily reacts with nucleophiles by the Michael reaction, this makes it quite easy to obtain bipharmacophoric compounds on their basis [14]. For this purpose, various amines [15,16] such as fragments of drugs can be used, for example, the conjugates of lactones with anthracycline antibiotics which show both increased efficiency and reduced cardiotoxicity [17]. Interesting derivatives are described in [18], where the sesquiterpene lactone parthenolide was combined in one molecule with the drugs cytarabine and melphalan. Bipharmacophore derivatives ultimately showed higher efficacy against tumor cell lines than the original substances. Thus, the structural modification of sesquiterpene lactones by obtaining the bipharmacophoric conjugates is a promising direction in the development of new effective drugs.

Proceeding with the research related to the construction of bipharmacophore compounds using natural substrates [19,20,21], we report on the synthesis of conjugates of sesquiterpene lactones: dehydrocostuaslactone and alantolactone with polyalkoxybenzenes and the study of their cytotoxic properties. Interest in the selected objects is due to the fact that alantolactone, being one of the actively studied eudesmane-type sesquiterpene lactones, has a wide pharmacological profile, including antitumor activity on neoplastic cells [22]. High cytotoxicity was also found for dehydrocostuslactone and some of its derivatives [23,24]. In turn, polyalkoxybenzenes are widespread pharmacophores that are part of various natural compounds with a wide range of biological activity, including antiproliferative [25,26,27]. Therefore, the synthesis of conjugates based on sesquiterpene lactones and polyalkoxybenzenes seems to be a promising strategy aimed at the development of bipharmacophoric compounds with cytotoxic properties.

**Figure 1:**
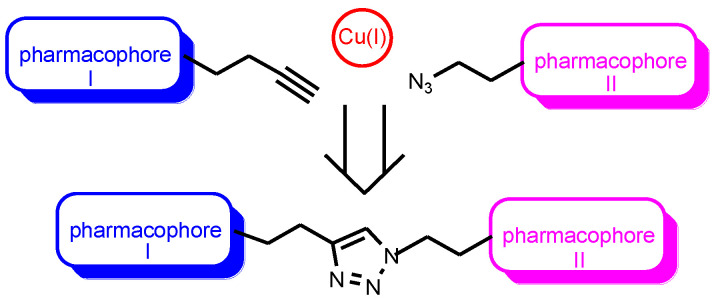
Copper-catalyzed cycloaddition reaction of organic azides to terminal acetylene-“click” reaction.

A copper-catalyzed “click” reaction of cycloaddition of organic azides to terminal acetylenes was proposed by Professor Sharpless and is used for the construction of biologically active conjugates [28,29,30] (Figure 1).

The reaction is very simple and provides a practically quantitative yield of the target compounds, which is widely recognized among organic chemists. In 2022, its authors were awarded the Nobel Prize. Currently, “click” reaction is the main tool of medicinal chemistry in the synthesis of conjugates with biological properties. It is important to note that the formed 1,2,3-triazole heterocyclic spacer, as a result of the [2 + 3]-cycloaddition reaction, is an additional pharmacophore fragment that affects the pharmacological profile of the conjugate [31,32]. The conjugation of two biologically active substrates or pharmacophores by “click” chemistry involves their preliminary functionalization, i.e., introduction of terminal acetylene and azide group.

## 2. Results and Discussion

### 2.1. Chemistry

**Scheme 1:**
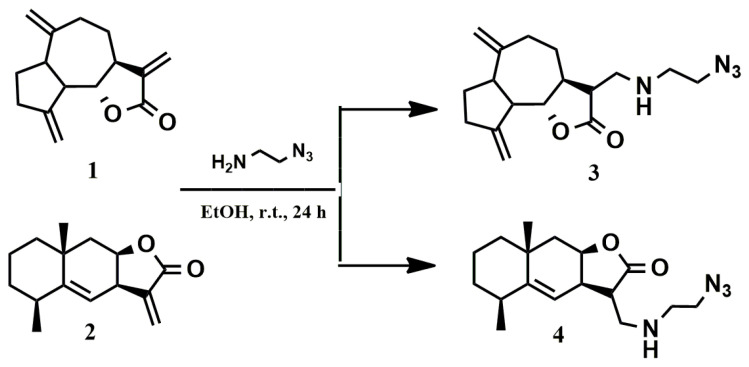
Introduction of the azide group into dehydrocostuslactone and alantolactone during the addition reaction at *exo*-methylene fragments.

To introduce the azide group into dehydrocostuslactone (**1**) and alantolactone (**2**) structures, we used 2-azidoethylamine, which easily adds to the activated *exo*-methylene fragments of lactones **1** and **2** (Scheme 1). The reaction was carried out at room temperature using ethanol as solvent. The course of the reaction was monitored using thin layer chromatography. The reaction proceeds in an almost quantitative yield. After the removal of the solvent, functionalized lactones **3** and **4**, as a mixture of isomers (ratio 1:1), were further used without purification. Analytically pure samples of **3** and **4** were obtained after column chromatography on silica gel (eluent: petroleum ether/ethyl acetate, 1:1) with good yields (82% for **3** and 85% for **4**).

**Scheme 2:**
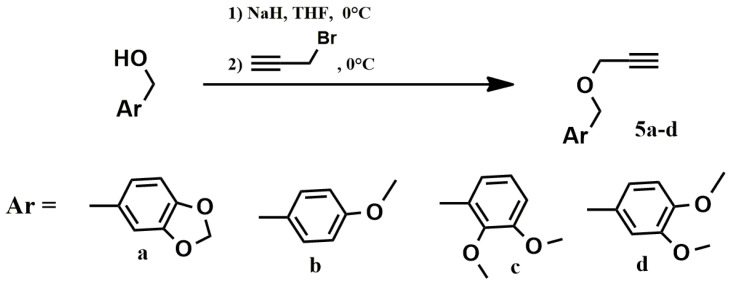
Preparation of benzylpropargyl ethers **5a**–**d**.

Benzylpropargyl ethers **5a**–**d** were used as the second, acetylene block, to implement the “click” reaction, the synthesis of which was performed by alkylation of the corresponding benzyl alcohols with propargyl bromide (Scheme 2).

**Scheme 3:**
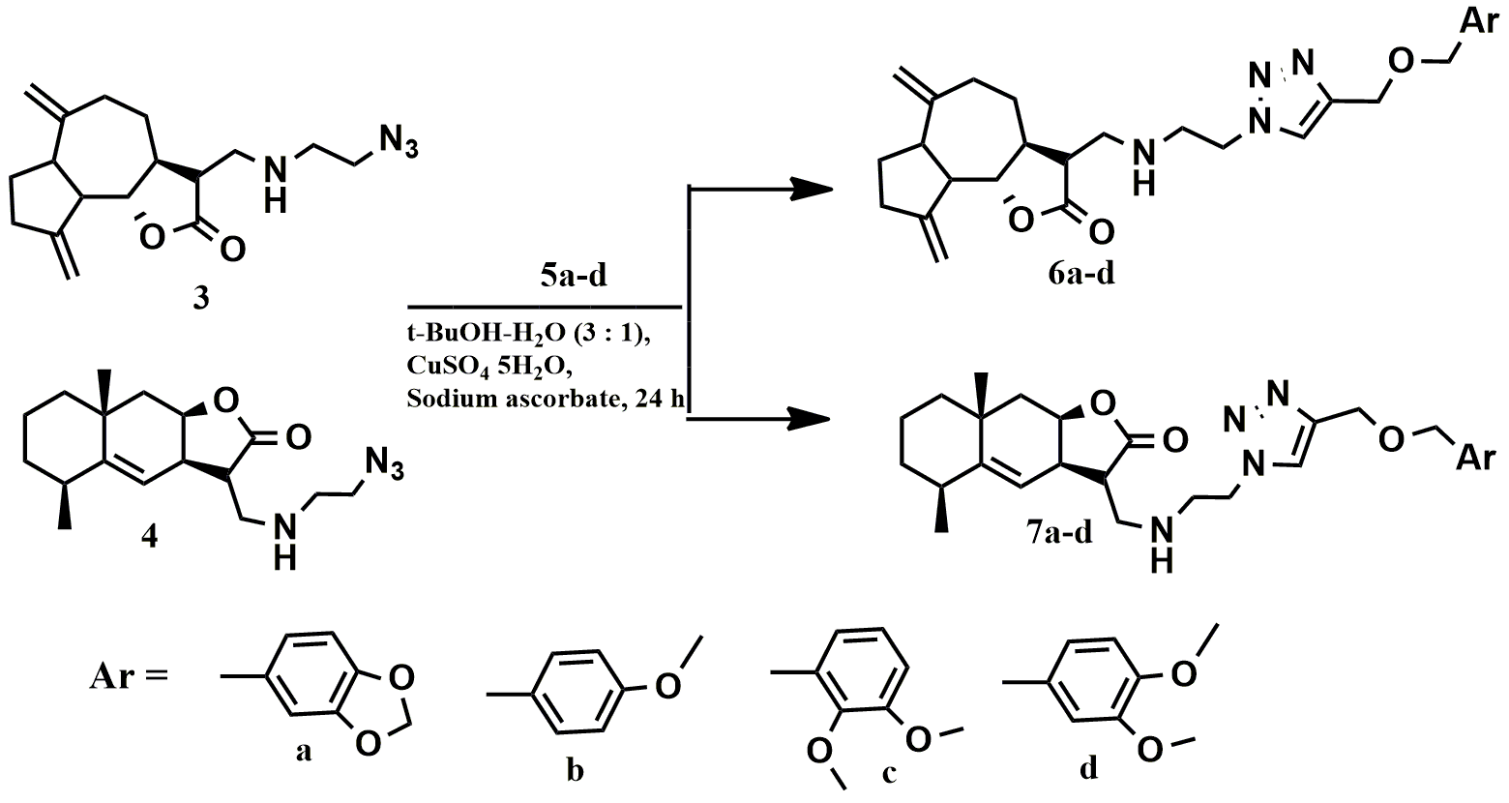
[2 + 3]-cycloaddition reaction of azides **3** and **4** to acetylenes **5a**–**d**.

The [2 + 3]-cycloaddition of azides **3** and **4** to acetylenes **5a**–**d** was carried out in accordance with the previously chosen conditions for the “click” reaction (Scheme 3) [33]. The course of the reaction was monitored using thin layer chromatography. Target conjugates **6a**–**d** and **7a**–**d**, which are viscous oils, were isolated by column chromatography. The composition of compounds **6a**–**d** and **7a**–**d** was established by elemental analysis and mass spectrometry, and the structure was proved by IR, ^1^H, ^13^C NMR spectroscopy (Supplementary materials). The main criteria indicating the formation of trizole ring were observed in all spectra. In the ^1^H NMR spectra of the reaction mass there are signals in the region of 7.64–7.70 ppm, characteristic of the =C-H proton of the triazole heterocycle. The ^13^C NMR spectra contained signals at 123.06–123.19 and 144.59–144.97 ppm which could be assigned to the carbon atoms of the 1,2,3-triazole ring.

**Scheme 4:**
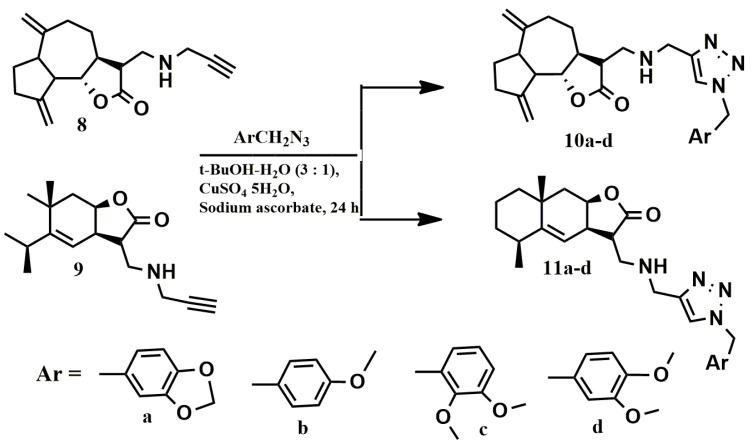
Scheme for obtaining conjugates **10a**–**d** and **11a**–**d**.

The biological properties of the obtained conjugates **6a**–**d** and **7a**–**d** depend on the the nature of the spacer connecting the lactone fragment with the benzyl moieties. In order to better understand the effect of this spacer, we synthesized conjugates **10a**–**d** and **11a**–**d** described earlier (Scheme 4) [34].

The next stage of our work consisted of studying the biological properties of the target conjugates of dehydrocostuslactone and allantolactone **6a**–**d**, **7a**–**d**, **10a**–**d** and **11a**.

### 2.2. Biological Evaluation

As mentioned above, the compounds containing pharmacophore fragments of lactones and polyalkoxybenzenes can act as potential antitumor chemotherapeutic agents. Therefore, from the point of view of a biological experiment, it was advisable to study the effect of synthesized compounds on the survival of various tumor and normal cells. In order to elucidate the possible mechanisms of the toxic action of the obtained conjugates, we analyzed their influence on the functioning of mitochondria and the glycolytic profile of the tumor cells. Moreover, based on the results of the experiments, it seemed interesting to evaluate the structure-activity relationships, especially the contribution of the nature of the spacer and lactone to the antitumor potential.

To assess the cytotoxic profile of the synthesized bipharmacophore molecules, a number of cell lines of tumor origin (SH-SY5Y, HeLa, Hep-2, A549) were used. Cells of normal origin Hek 293 were used to compare the effect of substances on the transformed and healthy microenvironment. A well-known natural antitumor drug, arglabin, a sesquiterpene lactone isolated from *Artemisia glabella*, was used as a reference compound.

Table 1 shows the IC_50_ values of the cytotoxic effect (the concentrations at which 50% cell death occurs). Among the compounds containing in their structure a longer spacer between two pharmacophores, the substances based on alantolactone showed the highest activity. At the same time, judging by the cytotoxicity indicators for all cell lines, compound **7a** proved to be the most effective; its IC_50_ values varied from 29 to 55 μM. It is also worth noting that a similar level of cytotoxicity was observed on healthy Hek 293 cells.

Interestingly, in the group of substances with a short spacer, the most pronounced cytotoxicity was shown by the compounds containing another natural fragment: dehydrocostus lactone, and the level of cytotoxic action for compounds **10a** and **10b** against SH-SY5Y and HeLa tumor cells exceeded that of arglabin, for which the value of IC_50_ did not go beyond 20 µM. Moreover, for these compounds, a reduced toxic effect was observed in relation to the cell line of normal origin Hek 293. A similar trend was observed for arglabin.

Thus, summarizing the results obtained in the study of the cytotoxic profile of the synthesized compounds, several key points can be distinguished: (1) when the spacer is elongated, the compounds based on alantolactone **7a**–**7d** increase their cytotoxic properties, while the selectivity of action against tumor cells is not observed; (2) for the substances containing dehydrocostuslactone, most active are the compounds with a short spacer **10a**–**d**, which selectively and most effectively suppressed the survival of tumor cells, similarly to arglabin. It should also be noted that the highest efficiency was observed in compounds containing benzylpropargyl ethers **a** and **b** as the second pharmacophore.

When studying the potential mechanisms of the cytotoxic action of the synthesized substances, their influence on several processes that play an important role in the functioning of tumor cells was determined.

**Figure 2:**
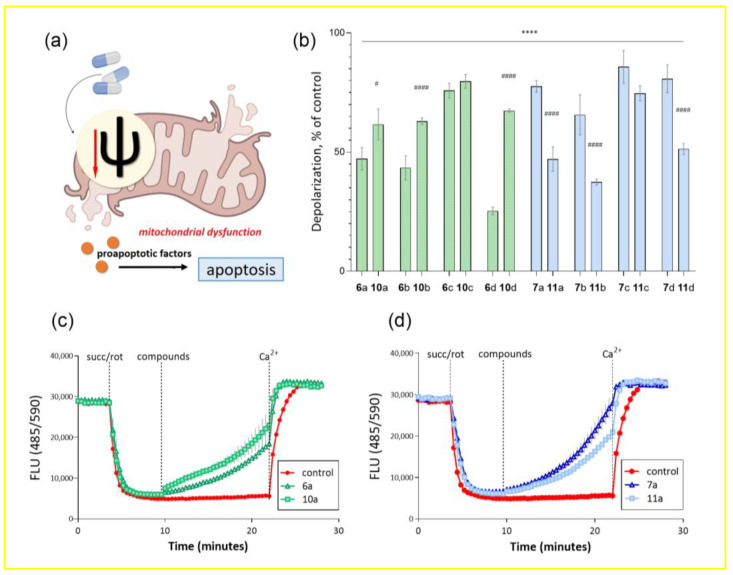
Effect of conjugates of dehydrocostuslactone and alantolactone with alkoxy-substituted benzyl azides on the transmembrane potential of isolated rat liver mitochondria. (**a**) Schematic representation of the effect of mitochondrial membrane depolarization on triggering cell death via the mitochondrial-dependent apoptosis pathway. (**b**) Percentage of mitochondrial membrane depolarization at a given time interval relative to control (as mean ± SEM (*n* = 3)). **** *p* ≤ 0.0001 compared to control, # *p* ≤ 0.05 and #### *p* ≤ 0.0001 compared to corresponding long spacer conjugate (one-way ANOVA). Kinetic curves of changes in the transmembrane potential of mitochondria under the action of conjugates (**c**)—dehydrocostuslactone **6a** and **10a** and (**d**)—alantolactone **7a** and **11a** with aromatic alkoxy derivatives. The concentration of the studied compounds was 100 μM, potassium succinate, 5 μM, rotenone, 0.5 μM, Ca^2+^, 25 μM.

It is well known that there are two main apoptotic pathways of programmed cell death: death receptor-mediated extrinsic and mitochondrial intrinsic pathways of apoptosis [36]. Loss of the mitochondrial membrane potential (ΔΨm) has been reported to lead to the induction of apoptosis via the mitochondrial pathway [37]; it is an early event preceding cell death. It has been proven that this may be due to the following cascade of events: depolarization of the mitochondrial membrane causes the association of proteins that are part of the mitochondrial permeability transition pore (in particular, adenine nucleotide translocator 1 in the inner mitochondrial membrane and matrix locating cyclophilin-D), triggering its opening. This leads to the depletion of ATP and release of proapoptotic factors into the cytosol, activation of caspases and, as a result, triggering of cell death (Figure 2a) [38,39]. Therefore, a promising direction in the search for anticancer drugs is the synthesis of compounds that can lead to depolarization of the mitochondrial membrane. For example, a decrease in the transmembrane potential under the action of therapeutic agents in death-resistant cells of malignant neoplasm, prostate carcinoma, induces apoptosis through the mitochondrial pathway [40].

ΔΨm was estimated from fluorescence quenching of the lipophilic cationic dye safranin A due to its accumulation inside energized mitochondria. Figure 2b shows the percentage of mitochondrial membrane depolarization induced by the test compounds. It can be seen that all substances to some extent led to a decrease in the membrane potential of isolated rat liver mitochondria. There is also a clear correlation between the percentage of depolarization and the cytotoxicity shown earlier for substances with different lactones and spacer lengths. In the case of allantolactone-containing compounds, substances **7a**–**d** led to almost complete depolarization of the mitochondrial membrane, while for substances with a short spacer this activity was lower. For dehydrocostus-containing substances, a pronounced difference was observed only for compounds **6d** and **10d**, where the compound with a short spacer had the highest efficiency.

As an example, for compounds **6a**, **10a** and **7a**, **11a**, Figure 2c,d show the kinetic curves of changes in the fluorescence of a mitochondrial suspension over time after the addition of modulators for a more visual presentation of the results. Thus, when succinate and rotenone were added, mitochondrial membrane energization and a decrease in the fluorescent response of the voltage-dependent label of safranin A were observed. Against this background, the test compounds were added, and a reaction was observed over a certain period of time accompanied by an increase in fluorescence in the samples and no changes in the control. The 100% depolarization of the mitochondrial membrane was observed upon the addition of calcium ions, which induce the opening of mitochondrial pores and lead to energetic collapse of organelles.

Thus, the property of the synthesized compounds to lead to transmembrane potential dissipation and energetic collapse of mitochondria can serve as a probable mechanism leading to the triggering of a cascade of tumor cell death via the apoptosis pathway and cause the previously detected cytotoxicity.

**Figure 3:**
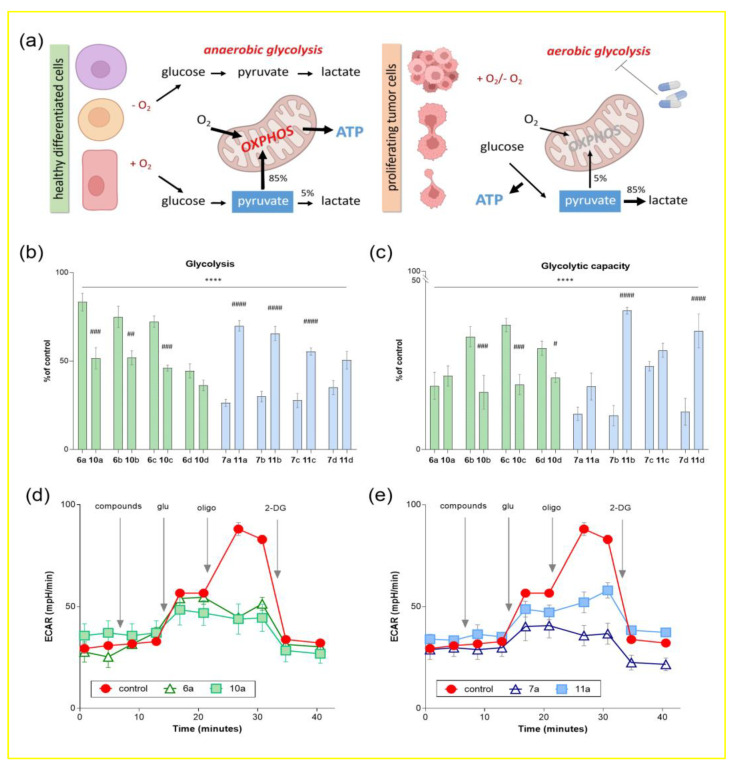
Influence of conjugates of dehydrocostuslactone and alantolactone with alkoxy-substituted benzyl azides on the process of glycolysis of the tumor neuroblastoma cell line SH-SY5Y. (**a**) Schematic representation of differences in the metabolism of normal and tumor cells. An increase in the rate of glucose uptake and preferential production of lactate is defined as the Warburg Effect. The influence of the studied compounds on the main calculated parameters of the glycolytic function of SH-SY5Y: (**b**) glycolysis (the difference between the extracellular acidification rate (ECAR) with the addition of glucose and basal glycolysis); (**c**) glycolytic capacity (the difference between the rate of extracellular acidification of the medium with the addition of the ATP synthase blocker oligomycin and basal glycolysis). Data are presented as a percentage relative to control (as mean ± SEM (*n* = 3)). **** *p* ≤ 0.0001 compared to control, # *p* ≤ 0.05, ## *p* ≤ 0.01, ### *p* ≤ 0.001 and #### *p* ≤ 0.0001 compared to the corresponding long spacer conjugate (one-way ANOVA). Kinetic curves of changes in the glycolytic profile under the action of conjugates (**d**) dehydrocostuslactone **6a** and **10a** and (**e**) alantolactone **7a** and **11a** with aromatic alkoxy derivatives. The concentration of the studied compounds was 100 µM, glucose was 10 mM, oligomycin was 1 µM, and 2-fluoro-2-deoxy-D-glucose was 25 mM.

It is well known that the metabolism of tumor cells is fundamentally different from that of normal cells and is characterized by greater activity, which ensures a higher proliferation rate and the ability to prevent cell death. Regardless of abundant oxygen availability, tumor cells predominantly use glycolysis for energy production mechanisms from lactic acid fermentation and have an extremely high rate of this process, in contrast to the normal ones with predominant energy production due to oxidative phosphorylation (Figure 3a) [41,42]. This phenomenon is called the Warburg effect, first described by the German biochemist Otto Warburg in 1920 [43]. Aerobic glycolysis is a well-known feature of the metabolism of transformed cells, and it can be considered as a promising drug target in the treatment of oncopathologies [44]. For example, it has been shown that blocking glycolysis in melanoma cells reduces ATP levels and inhibits cell proliferation [45].

Using the cell metabolism analyzer Agilent Seahorse XF96e Analyzer (Seahorse Bioscience, Billerica, MA, USA), we assessed the extracellular acidification rate (ECAR) in cells of tumor origin SH-SY5Y as an indicator of glycolysis. The effect of compounds on the key parameters of glycolytic function, glycolysis and glycolytic capacity, was analyzed by monitoring changes in ECAR in response to sequential addition of modulators.

Figure 3b,c shows the percentage change in ECAR relative to controls containing the equivalent volume of solvent for the test substances (DMSO ≤ 1%). It is evident that in SH-SY5Y the cells treated with 100 μM concentration of all dehydrocostuslactone and allantolactone conjugates with alkoxy-substituted benzyl azides, both parameters of glycolytic function were significantly reduced, including basal glycolysis and glycolytic capacity, reaching a minimum after the injection of dehydrocostuslactone-based compounds **6d** and **10a**–**d** and alantolactone **7a**–**d** (in the case of glycolysis), as well as **6a**, **10a**–**d** and **7a**–**d**, **11a** (in the case of glycolytic capacity). Moreover, in this experiment, a similar trend is observed as in the study of the effect of the synthesized compounds on the transmembrane potential, where the correlation between the structure of the compounds and their activity is clearly visible. Again, in the case of allantolactone-containing conjugates, long spacer compounds **7a**–**d** resulted in significantly more effective inhibition of glycolysis parameters than short spacer compounds **11a**–**d**. In turn, in the case of dehydrocostus-containing conjugates, compounds with a short spacer **6a**–**d** had the highest efficiency.

As an example, for compounds **6a**, **10a** and **7a**, **11a** and better visualizing of the results obtained, Figure 3d,e shows the kinetic curves of ECAR changes in cells over time, as modulators are added. Thus, glucose injection led to a sharp increase in this indicator, while the cells pretreated with the studied compounds showed ECAR suppression. A similar situation was also observed in the case when the addition of oligomycin in the control samples showed the maximum glycolytic activity. In turn, under the action of **6a**, **10a** and **7a**, **11a**, a decrease in ECAR by more than 50% was noted.

Thus, the ability of conjugates of dehydrocostuslactone and alantolactone with alkoxy-substituted benzyl azides to suppress the parameters of the glycolytic function of cells of tumor origin can also be considered as a possible mechanism of their previously shown cytotoxic activity.

### 2.3. Evaluation of Synthesized Compounds Pharmacokinetic Parameters

For potential drugs, an important step is to predict their physicochemical properties. Additionally, for the tested compounds, their pharmacokinetic parameters (ADME/Tox) were determined by computer analysis using the ‘QikProp’ subroutine. This program allows you to predict more than 40 different parameters, some of which are presented in Table 2.

By analyzing the values of “#stars” it is possible to identify structures that can be positioned as potential drugs. The following descriptors are included in the definition of “#stars”: values of molecular masses, dipole moments, electron affinity and ionization potential; volume characteristics of the molecule, the number of rotational bonds and averaged values of the number of donors and acceptors of hydrogen bonds; values characterizing lipophilicity, solubility, permeability through the blood–brain barrier, affinity for albumin, as well as the number of possible metabolic reactions that the compounds under study can enter. According to this parameter and the totality of calculated data, in general, all compounds can be positioned as potential drugs that meet the criteria of “drug-like” with a high degree of permeability through the blood–brain barrier, satisfying lipophilicity coefficient and good predicted oral absorption.

## 3. Materials and Methods

### 3.1. Reagents and Materials

All commercial reagents were used as purchased without further purification, and all solvents used in the reactions were freshly distilled from appropriate drying agents before use. Analytical TLC was performed on Merck silica gel 60 F_254_ plates (Darmstadt, Germany), visualized under UV light (λ_max_ = 254 nm), or by staining with iodine vapor. Column chromatography was carried out using Merck silica gel (Kieselgel 60, 0.063–0.200 mm, Darmstadt, Germany) and petroleum ether/ethyl acetate, petroleum ether/acetone, dichloromethane/ethanol as an eluent. NMR spectra were recorded with Bruker AV-400 spectrometer (^1^H, 400.13 and ^13^C, 100.61 MHz) using residual proton signals of deuterated solvent as an internal standard rel. to TMS. The ^13^C NMR spectra were registered using the JMODECHO mode; the signals for the C-atom bearing odd and even numbers of H-atoms have opposite polarities. IR spectra were recorded in KBr pellets on a Fourier-spectrometer “Magna-IR750” (Nicolet, QC, Canada), resolution 2 cm^−1^, 128 scans. Liquid chromato–mass spectrometry (HPLC-MS) was performed using a Shimadzu LCMS-2020 instrument (Kyoto, Japan) by means of electrospray ionization (ESI). The range of detected masses was of *m*/*z* 50 to 2000, the measurements were performed in the positive ions mode (voltage at the interface 4500 V and voltage at the detector 1000 V). Acetonitrile (high-purity grade) was used as the mobile phase. Analytical data (C, H, N content) were obtained with a Carlo Erba model 1106 microanalyzer.

Dehydrocostus lactone (**1**) and alantolactone (**2**) were isolated from plant substrates according to previously reported procedures [46,47].

1-Amino-2-azidoethane was obtained according to known procedure [48].

The synthesis methods and physicochemical characteristics of propargylated lactones **8,9** and their 1,2,3-triazole derivatives **10,11** have been described earlier [34].

### 3.2. General Procedure for the Synthesis of Azides (***3**, **4***)

To a solution of lactone **1** or **2** (2 mmol, 1.0 eq) in EtOH (7 mL) 1-amino-2-azidoethane (4 mmol, 2.0 eq) was added. The mixture was stirred for 24 h at room temperature. The solvent was evaporated affording azide (quant) as viscous yellow oil, which was used for the next step without further purification. Analytical grade sample was obtained by column chromatography (pet. ether/ethyl acetate, 1:1).

(3R,3aS,9bS)-3-(((2-Azidoethyl)amino)methyl)-6,9-dimethylenedecahydroazuleno [4,5-b]furan-2(9bH)-one (**3**)

Yellow oil (82%). ^1^H NMR (400 MHz, CDCl3) δ 5.30 (1H, s, H-13a), 5.17 (1H, s, H-13b), 4.87 (1H, s, H-14a), 4.76 (1H, s, H-14b), 3.96 (1H, t, *J* = 9.1 Hz, H-2), 3.37 (2H, t, *J* = 5.6 Hz, H_2_-17), 2.97 (1H, dd, *J* = 12.1 Hz, *J* = 3.8 Hz, H-15a), 2.89–2.81 (4H, m, H-1, H-7, H-12, H-15b), 2.51–2.45 (3H, m, H-5a, H-9), 2.39–2.34 (2H, m, H_2_-16), 2.15–1.85 (5H, m, H-3, H-4a, H-5b, H-8), 1.39–1.24 (1H, m, H-4b). ^13^C NMR (100 MHz, CDCl_3_) δ 177.51 (C-11), 151.56 (C-10), 149.65 (C-6), 111.64 (C-13), 108.85 (C-14), 85.62 (C-2), 51.64 (C-12), 50.96 (C-17), 48.82 (C-15), 47.30 (C-1), 47.11 (C-16), 46.74 (C-7), 44.57 (C-3), 37.46 (C-9), 32.40 (C-5), 32.33 (C-4), 29.95 (C-8). IR (KBr) *v*_max_ 2929, 2100, 1767, 1456, 1340, 1176, 1004, 894 cm^−1^. Anal. Calc. for C_17_H_24_N_4_O_2_·0.1 CH_2_Cl_2_: C, 63.22; H, 7.51; N, 17.24%. Found: C, 63.15; H, 7.42; N, 17.09%.

(3S,3aR,5S,8aR,9aR)-3-(((2-Azidoethyl)amino)methyl)-5,8a-dimethyl-3,3a,6,7,8,8a,9,9a-octahydronaphtho [2,3-b]furan-2(5H)-one (**4**).

Yellow oil (85%). ^1^H NMR (400 MHz, CDCl_3_) δ 5.15 (1H, d, *J* = 2.8 Hz, H-7), 4.76 (1H, m, H-9), 3.44 (2H, d, *J* = 5.6 Hz, H_2_-17), 3.16–3.13 (1H, m, H-8), 3.02–2.78 (5H, m, H-11, H_2_-15, H_2_-16), 2.50–2.47 (1H, m, H-3), 2.11 (1H, dd, *J* = 14.6 Hz, *J* = 3.1 Hz, H-10a), 1.87–1.76 (1H, m, H-2a), 1.61–1.42 (6H, m, H-1, H-2b, H-6, H-10b), 1.23 (3H, s, H_3_-13), 1.12 (3H, d, *J* = 7.6 Hz, H_3_-14). ^13^C NMR (100 MHz, CDCl_3_) δ 177.12 (C-12), 150.10 (C-4), 114.58 (C-7), 76.69 (C-9), 50.44 (C-17), 47.93 (C-15), 45.54 (C-16), 45.02 (C-11), 42.04 (C-10), 41.50 (C-6), 37.77 (C-3), 36.92 (C-8), 32.31 (C-5), 32.15 (C-2), 27.99 (C-13), 22.32 (C-14), 16.18 (C-1). IR (KBr) *v*_max_ 2929, 2101, 1762, 1457, 1340, 1180, 1151, 1039, 733 cm^−1^. Anal. Calc. for C_17_H_26_N_4_O_2_·0.15 CH_2_Cl_2_: C, 62.20; H, 8.00; N, 16.92%. Found: C, 62.68; H, 8.03; N, 16.80%.

### 3.3. General Procedure for the Synthesis of Alkynes (***5a**–**d***)

To a stirred solution of alcohol (7 mmol, 1.0 eq) in anhydrous THF (10 mL) was added sodium hydride (60 percent in oil, 7 mmol, 1.0 eq) at 0 °C. The mixture was stirred for 30 min and propargyl bromide (80% in toluene, 7 mmol, 1 eq) was then added dropwise at 0 °C. Stirring was continued at room temperature overnight. The solvent was evaporated, and the residue was extracted with ethyl acetate (3 × 25 mL). The organic layer was washed with brine, dried over Na_2_SO_4_ and concentrated in vacuo. The crude residue was purified by column chromatography (pet. ether/acetone, 100:2) to give the desired product as a colorless oil. Spectral data of the known compounds **5a** [33], **5b** [49], and **5d** [50] fit well the literature data.

1,2-Dimethoxy-3-((prop-2-yn-1-yloxy)methyl)benzene (**5c**).

Viscous yellow oil (85%). ^1^H NMR (400 MHz, CDCl_3_) δ 7.08 (1H, t, *J* = 8.0 Hz, H-5), 7.02 (1H, d, *J* = 8.0 Hz, H-6), 6.91 (1H, d, *J* = 8.0 Hz, H-4), 4.69 (2H, s, OCH_2_Ar), 4.23 (2H, d, *J* = 2.4 Hz, OCH_2_C≡), 3.89, 3.88 (6H, both s, OCH_3_), 2.49 (1H, t, *J* = 2.4 Hz, CH). ^13^C NMR (100 MHz, CDCl_3_) δ 152.50 (C-2), 147.21 (C-1), 131.14 (C-3), 123.88 (C-4), 121.37 (C-5), 112.06 (C-6), 79.67 (C≡), 74.31 (≡CH), 66.33 (OCH_2_), 60.97 (OCH_3_), 57.19 (OCH_2_), 55.62 (OCH_3_). Anal. Calc. for C_12_H_14_O_3_·0.5 (CH_3_)_2_CO: C, 68.64; H, 7.38%. Found: C, 68.92; H, 7.28%.

### 3.4. General Procedure for the “Click” Reactions

To a stirred solution of azide (0.3 mmol, 1.0 eq) in t-BuOH—H_2_O (3:1, 4 mL), CuSO_4_·5H_2_O (0.015 mmol, 5 mol.%), sodium ascorbate (0.03 mmol, 10 mol.%), and the corresponding alkyne (0.315 mmol, 1.05 eq.) were added. The reaction mixture was stirred for 24 h at room temperature (TLC monitoring). The solvent was removed in vacuo, and the remaining crude product was purified by column chromatography (dichloromethane/ethanol, 100:0.2 to 100:5) to afford the corresponding product as a viscous yellow oil.

(3R,3aS,9bS)-3-(((2-(4-((Benzo[d][1,3]dioxol-5-ylmethoxy)methyl)-1H-1,2,3-triazol-1-yl)ethyl)amino)methyl)-6,9-dimethylenedecahydroazuleno [4,5-b]furan-2(9bH)-one (**6a**).

Yellow oil (82%). ^1^H NMR (400 MHz, CDCl_3_) δ 7.66 (1H, s, H-18), 6.86 (1H, s, H-23) 6.81 (1H, d, *J* = 7.8 Hz, H-27), 6.76 (1H, d, *J* = 7.8 Hz, H-26), 5.94 (2H, s, H_2_-28), 5.16 (1H, s, H-13a), 5.03 (1H, s, H-13b), 4.86 (1H, s, H-14a), 4.75 (1H, s, H-14b), 4.64 (2H, s, H_2_-21), 4.49 (2H, s, H_2_-20), 4.45–4.42 (2H, m, H_2_-17), 3.93 (1H, t, *J* = 9.4 Hz, H-2), 3.11 (2H, t, *J* = 5.9 Hz, H_2_-16), 2.91 (1H, dd, *J* = 12.2 Hz, *J* = 4.2 Hz, H-15a), 2.85–2.73 (3H, m, H-7, H-12, H-15b), 2.52–2.42 (3H, m, H-1, H-9), 2.34–2.30 (1H, m, H-5a), 2.21–2.18 (1H, m, H-4a), 2.05–1.82 (4H, m, H-3, H-5b, H-8), 1.34–1.24 (1H, m, H-4b). ^13^C NMR (100 MHz, CDCl_3_) δ 177.47 (C-11), 151.54 (C-10), 149.57 (C-6), 147.53 (C-25), 146.98 (C-24), 144.77 (C-19), 131.43 (C-22), 123.11 (C-18), 121.39 (C-27), 111.66 (C-13), 108.85 (C-14), 108.43 (C-26), 107.86 (C-23), 100.78 (C-28), 85.59 (C-2), 72.13 (C-21), 63.21 (C-20), 51.59 (C-12), 49.90 (C-17), 49.07 (C-15), 47.29 (C-1), 46.71 (C-7), 46.69 (C-16), 44.56 (C-3), 37.37 (C-9), 32.32 (C-5), 32.30 (C-4), 29.93 (C-8). IR (KBr) *v*_max_ 2925, 1763 (C=O), 1491, 1251, 1040, 911, 809, 733 cm^−1^. Mass spectrum (LC-MS ESI), *m*/*z* (*I*_rel_, %): 529.35 (100) [M + Na]^+^. Anal. Calc. for C_28_H_34_N_4_O_5_·1.3 CH_2_Cl_2_: C, 57.04; H, 5.98; N, 9.08%. Found: C, 56.82; H, 6.57; N, 8.85%.

(3R,3aS,9bS)-3-(((2-(4-(((4-Methoxybenzyl)oxy)methyl)-1H-1,2,3-triazol-1-yl)ethyl)amino)methyl)-6,9-dimethylenedecahydroazuleno [4,5-b]furan-2(9bH)-one (**6b**).

Yellow oil (73%). ^1^H NMR (400 MHz, CDCl_3_) δ 7.69 (1H, s, H-18), 6.81 (2H, d, *J* = 8.4 Hz, H-23, H-27), 6.86 (2H, d, *J* = 8.4 Hz, H-24, H-26), 5.29 (1H, s, H-13a), 5.15 (1H, s, H-13b), 4.85 (1H, s, H-14a), 4.74 (1H, s, H-14b), 4.63 (2H, s, H_2_-21), 4.52 (2H, s, H_2_-20), 4.42–4.40 (2H, m, H_2_-17), 3.91 (1H, t, *J* = 9.4 Hz, H-2), 3.78 (3H, s, H_3_-28), 3.09 (2H, t, *J* = 5.8 Hz, H_2_-16), 2.90 (1H, dd, *J* = 12.2 Hz, *J* = 4.0 Hz, H-15a), 2.84–2.75 (3H, m, H-7, H-12, H-15b), 2.52–2.44 (3H, m, H-1, H-9), 2.32–2.30 (1H, m, H-5a), 2.20–2.17 (1H, m, H-4a), 2.04–1.83 (4H, m, H-3, H-5b, H-8), 1.33–1.19 (1H, m, H-4b). ^13^C NMR (100 MHz, CDCl_3_) δ 177.41 (C-11), 158.99 (C-25), 151.51 (C-10), 149.53 (C-6), 144.80 (C-19), 129.62 (C-22), 129.31 (C-24, C-26), 123.06 (C-18), 113.53 (C-23, C-27), 111.59 (C-13), 108.77 (C-14), 85.53 (C-2), 71.90 (C-21), 63.07 (C-20), 55.00 (C-28), 51.55 (C-12), 49.84 (C-17), 49.01 (C-15), 47.23 (C-1), 46.64 (C-7), 46.62 (C-16), 44.48 (C-3), 37.34 (C-9), 32.27 (C-5), 32.24 (C-4), 29.85 (C-8). IR (KBr) *v*_max_ 2930, 1767 (C=O), 1613, 1515, 1248, 1175, 1034, 822, 734 cm^−1^. Mass spectrum (LC-MS ESI), *m*/*z* (*I*_rel_, %): 493.40 (50) [M + H]^+^. Anal. Calc. for C_28_H_36_N_4_O_4_·0.5 CH_2_Cl_2_: C, 63.97; H, 6.97; N, 10.47%. Found: C, 63.91; H, 6.78; N, 10.41%.

(3R,3aS,9bS)-3-(((2-(4-(((2,3-Dimethoxybenzyl)oxy)methyl)-1H-1,2,3-triazol-1-yl)ethyl)amino)methyl)-6,9-dimethylenedecahydroazuleno [4,5-b]furan-2(9bH)-one (**6c**).

Yellow oil (81%). ^1^H NMR (400 MHz, CDCl_3_) δ 7.67 (1H, s, H-18), 7.04 (1H, t, *J* = 7.8 Hz, H-26), 7.00 (1H, dd, *J* = 7.6 Hz, *J* = 1.8 Hz, H-25), 6.89 (1H, dd, *J* = 7.6 Hz, *J* = 1.8 Hz, H-27), 5.15 (1H, br. s, H-13a), 5.02 (1H, br. s, H-13b), 4.85 (1H, s, H-14a), 4.74 (1H, s, H-14b), 4.70 (2H, s, H_2_-21), 4.64 (2H, s, H2-20), 4.44–4.41 (2H, m, H_2_-17), 3.92 (1H, t, *J* = 9.4 Hz, H-2), 3.85, 3.82 (6H, both s, H_3_-28, H_3_-29), 3.10 (2H, t, *J* = 5.9 Hz, H_2_-16), 2.90 (1H, dd, *J* = 12.2 Hz, *J* = 4.3 Hz, H-15a), 2.85–2.76 (3H, m, H-7, H-12, H-15b), 2.50–2.41 (3H, m, H-1, H-9), 2.33–2.30 (1H, m, H-5a), 2.21–2.16 (1H, m, H-4a), 2.05–1.82 (4H, m, H-3, H-5b, H-8), 1.30–1.22 (1H, m, H-4b). ^13^C NMR (100 MHz, CDCl_3_) δ 177.45 (C-11), 152.37 (C-23), 151.53 (C-10), 149.58 (C-6), 146.95 (C-24), 144.91 (C-19), 131.50 (C-22), 123.82 (C-27), 123.07 (C-18), 121.17 (C-26), 111.82 (C-25), 111.61 (C-13), 108.81 (C-14), 85.58 (C-2), 67.09 (C-21), 63.63 (C-20), 60.82 (C-28), 55.52 (C-29), 51.57 (C-12), 49.89 (C-17), 49.06 (C-15), 47.25 (C-1), 46.72 (C-16), 46.67 (C-7), 44.56 (C-3), 37.37 (C-9), 32.33 (C-5), 32.31 (C-4), 29.91 (C-8). IR (KBr) *v*_max_ 2934, 1765 (C=O), 1588, 1483, 1278, 1175, 1069, 1005, 895, 734 cm^−1^. Mass spectrum (LC-MS ESI), *m*/*z* (*I*_rel_, %): 523.45 (40) [M + H]^+^, 545.45 (100) [M + Na]^+^. Anal. Calc. for C_29_H_38_N_4_O_5_·0.6 CH_2_Cl_2_: C, 61.98; H, 6.89; N, 9.77%. Found: C, 62.39; H, 6.77; N, 9.11%.

(3R,3aS,9bS)-3-(((2-(4-(((3,4-Dimethoxybenzyl)oxy)methyl)-1H-1,2,3-triazol-1-yl)ethyl)amino)methyl)-6,9-dimethylenedecahydroazuleno [4,5-b]furan-2(9bH)-one (**6d**).

Yellow oil (71%). ^1^H NMR (400 MHz, CDCl_3_) δ 7.65 (1H, s, H-18), 6.88–6.85 (2H, m, H-23, H-27), 6.79 (1H, d, *J* = 8.0 Hz, H-26), 5.11 (1H, s, H-13a), 4.99 (1H, s, H-13b), 4.82 (1H, s, H-14a), 4.71 (1H, s, H-14b), 4.61 (2H, s, H_2_-21), 4.50 (2H, s, H_2_-20), 4.41–4.38 (2H, m, H_2_-17), 3.89 (1H, t, *J* = 9.5 Hz, H-2), 3.84, 3.83 (6H, both s, H_3_-28, H_3_-29), 3.07 (2H, t, *J* = 5.9 Hz, H_2_-16), 2.88 (1H, dd, *J* = 12.3 Hz, *J* = 4.2 Hz, H-15a), 2.81–2.70 (3H, m, H-7, H-12, H-15b), 2.49–2.38 (3H, m, H-1, H-9), 2.30–2.26 (1H, m, H-5a), 2.20–2.12 (1H, m, H-4a), 2.01–1.80 (4H, m, H-3, H-5b, H-8), 1.30–1.20 (1H, m, H-4b). ^13^C NMR (100 MHz, CDCl_3_) δ 177.36 (C-11), 151.45 (C-10), 149.43 (C-6), 148.62 (C-25), 148.30 (C-24), 144.59 (C-19), 129.99 (C-22), 123.11 (C-18), 120.26 (C-27), 111.54 (C-13), 110.94 (C-26), 110.54 (C-23), 108.68 (C-14), 85.47 (C-2), 72.10 (C-21), 62.97 (C-20), 55.57 (C-29), 55.53 (C-28), 51.48 (C-12), 49.74 (C-17), 48.95 (C-15), 47.14 (C-1), 46.57 (C-7), 46.53 (C-16), 44.39 (C-3), 37.26 (C-9), 32.19 (C-5), 32.15 (C-4), 29.80 (C-8). IR (KBr) *v*_max_ 2934, 1764 (C=O), 1516, 1265, 1139, 1028, 897, 733 cm^−1^. Mass spectrum (LC-MS ESI), *m*/*z* (*I*_rel_, %): 523.45 (50) [M + H]^+^, 545.45 (100) [M + Na]^+^. Anal. Calc. for C_29_H_38_N_4_O_5_·1.3 CH_2_Cl_2_: C, 57.49; H, 6.46; N, 8.85%. Found: C, 57.46; H, 6.89; N, 8.65%.

(3S,3aR,5S,8aR,9aR)-3-(((2-(4-((Benzo[d][1,3]dioxol-4-ylmethoxy)methyl)-1H-1,2,3-triazol-1-yl)ethyl)amino)methyl)-5,8a-dimethyl-3,3a,6,7,8,8a,9,9a-octahydronaphtho [2,3-b]furan-2(5H)-one (**7a**).

Yellow oil (78%). 1H NMR (400 MHz, CDCl_3_) δ 7.69 (1H, s, H-18), 6.86 (1H, s, H-23), 6.81 (1H, d, *J* = 8.0 Hz, H-27), 6.77 (1H, d, *J* = 8.0 Hz, H-26), 5.94 (2H, s, H_2_-28), 5.07 (1H, d, *J* = 2.8 Hz, H-7), 4.74–4.73 (1H, m, H-9), 4.64 (2H, s, H_2_-21), 4.49 (2H, s, H_2_-20), 4.48–4.43 (2H, m, H_2_-17), 3.24–3.18 (1H, m, H-8), 3.10–3.08 (2H, m, H_2_-16), 3.00–2.90 (2H, m, H_2_-15), 2.78–2.73 (1H, m, H-11), 2.47–2.44 (1H, m, H-3), 2.09 (1H, dd, *J* = 14.8 Hz, *J* = 3.2 Hz, H-10a), 1.85–1.77 (1H, m, H-2a), 1.60–1.41 (6H, m, H-1, H-2b, H-6, H-10b), 1.21 (3H, s, H_3_-13), 1.11 (3H, d, *J* = 7.6 Hz, H_3_-14). ^13^C NMR (100 MHz, CDCl_3_) δ 177.73 (C-12), 151.04 (C-4), 147.57 (C-25), 147.02 (C-24), 144.83 (C-19), 131.45 (C-22), 123.14 (C-18), 121.43 (C-27), 114.62 (C-7), 108.45 (C-26), 107.89 (C-23), 100.81 (C-28), 77.28 (C-9), 72.15 (C-21), 63.13 (C-20), 49.99 (C-17), 49.01 (C-15), 46.03 (C-16), 45.51 (C-11), 42.45 (C-10), 41.96 (C-6), 38.24 (C-3), 37.42 (C-8), 32.81 (C-5), 32.61 (C-2), 28.46 (C-13), 22.78 (C-14), 16.61 (C-1). IR (KBr) *v*_max_ 2928, 1758 (C=O), 1491, 1444, 1220, 1040, 929, 809, 735 cm^−1^. Mass spectrum (LC-MS ESI), *m*/*z* (*I*_rel_, %): 509.33 (100) [M + H]^+^. Anal. Calc. for C_28_H_36_N_4_O_5_·0.7 CH_2_Cl_2_: C, 60.68; H, 6.64; N, 9.86%. Found: C, 60.94; H, 6.41; N, 9.78%.

(3S,3aR,5S,8aR,9aR)-3-(((2-(4-(((4-Methoxybenzyl)oxy)methyl)-1H-1,2,3-triazol-1-yl)ethyl)amino)methyl)-5,8a-dimethyl-3,3a,6,7,8,8a,9,9a-octahydronaphtho [2,3-b]furan-2(5H)-one (**7b**).

Yellow oil (75%). ^1^H NMR (400 MHz, CDCl_3_) δ 7.68 (1H, s, H-18), 7.27 (2H, d, *J* = 8.6 Hz, H-23, H-27), 6.86 (2H, d, *J* = 8.5 Hz, H-24, H-26), 5.06 (1H, d, *J* = 2.8 Hz, H-7), 4.71–4.70 (1H, m, H-9), 4.63 (2H, s, H_2_-21), 4.52 (2H, s, H_2_-20), 4.47–4.44 (2H, m, H_2_-17), 3.78 (3H, s, H_3_-28), 3.21–3.15 (1H, m, H-8), 3.10–3.04 (2H, m, H_2_-16), 2.98–2.89 (2H, m, H_2_-15), 2.75–2.71 (1H, m, H-11), 2.46–2.43 (1H, m, H-3), 2.07 (1H, dd, *J* = 14.8 Hz, *J* = 3.0 Hz, H-10a), 1.85–1.75 (1H, m, H-2a), 1.59–1.39 (6H, m, H-1, H-2b, H-6, H-10b), 1.23 (3H, s, H_3_-13), 1.09 (3H, d, *J* = 7.6 Hz, H_3_-14). ^13^C NMR (100 MHz, CDCl_3_) δ 177.67 (C-12), 159.02 (C-25), 150.93 (C-4), 144.85 (C-19), 129.62 (C-22), 129.34 (C-24, 26), 123.10 (C-18), 114.60 (C-7), 113.55 (C-23, 27), 77.21 (C-9), 71.91 (C-21), 63.06 (C-20), 55.03 (C-28), 49.91 (C-17), 48.94 (C-15), 45.94 (C-16), 45.43 (C-11), 42.39 (C-10), 41.89 (C-6), 38.17 (C-3), 37.34 (C-8), 32.75 (C-5), 32.55 (C-2), 28.40 (C-13), 22.73 (C-14), 18.17 (C-1). IR (KBr) *v*_max_ 2929, 1759 (C=O), 1613, 1514, 1249, 1175, 1037, 822, 733 cm^−1^. Mass spectrum (LC-MS ESI), *m*/*z* (I_rel_, %): 495.45 (100) [M + H]^+^. Anal. Calc. for C_28_H_38_N_4_O_4_ 0.75 CH_2_Cl_2_: C, 61.85; H, 7.13; N, 10.03%. Found: C, 61.64; H, 7.07; N, 10.05%.

(3S,3aR,5S,8aR,9aR)-3-(((2-(4-(((2,3-Dimethoxybenzyl)oxy)methyl)-1H-1,2,3-triazol-1-yl)ethyl)amino)methyl)-5,8a-dimethyl-3,3a,6,7,8,8a,9,9a-octahydronaphtho [2,3-b]furan-2(5H)-one (**7c**).

Yellow oil (72%). ^1^H NMR (400 MHz, CDCl_3_) δ 7.70 (1H, s, H-18), 7.05 (1H, t, *J* = 7.8 Hz, H-26), 7.00 (1H, dd, *J* = 7.8 Hz, *J* = 1.7 Hz, H-25), 6.87 (1H, dd, *J* = 7.9 Hz, *J* = 1.7 Hz, H-27), 5.06 (1H, d, *J* = 3.1 Hz, H-7), 4.43–4.71 (1H, m, H-9), 4.70 (2H, s, H_2_-21), 4.64 (2H, s, H_2_-20), 4.49–4.42 (2H, m, H_2_-17), 3.85, 3.81 (6H, both s, H_3_-28, H_3_-29), 3.21–3.19 (1H, m, H-8), 3.10–3.05 (2H, m, H_2_-16), 2.97–2.92 (2H, m, H_2_-15), 2.78–2.73 (1H, m, H-11), 2.47–2.44 (1H, m, H-3), 2.08 (1H, dd, *J* = 14.8 Hz, *J* = 3.2 Hz, H-10a), 1.85–1.76 (1H, m, H-2a), 1.60–1.40 (6H, m, H-1, H-2b, H-6, H-10b), 1.22 (3H, s, H_3_-13), 1.10 (3H, d, *J* = 7.6 Hz, H_3_-14). ^13^C NMR (100 MHz, CDCl3) δ 177.73 (C-12), 152.40 (C-23), 151.01 (C-4), 146.98 (C-24), 144.97 (C-19), 131.48 (C-22), 123.86 (C-27), 123.13 (C-18), 121.23 (C-26), 114.59 (C-7), 111.87 (C-25), 77.28 (C-9), 67.11 (C-21), 63.63 (C-20), 60.85 (C-28), 55.55 (C-29), 49.93 (C-17), 48.99 (C-15), 45.98 (C-16), 45.45 (C-11), 42.43 (C-10), 41.95 (C-6), 38.22 (C-3), 37.38 (C-8), 32.79 (C-5), 32.59 (C-2), 28.44 (C-13), 22.76 (C-14), 16.59 (C-1). IR (KBr) *v*_max_ 2930, 1760 (C=O), 1588, 1483, 1278, 1176, 1069, 1008, 734 cm^−1^. Mass spectrum (LC-MS ESI), *m*/*z* (*I*_rel_, %): 525.50 (100) [M + H]^+^, 547.45 (50) [M + Na]^+^. Anal. Calc. for C_29_H_40_N_4_O_5_·1.15 CH_2_Cl_2_: C, 58.19; H, 6.85; N, 9.00%. Found: C, 58.26; H, 6.77; N, 8.79%.

(3S,3aR,5S,8aR,9aR)-3-(((2-(4-(((3,4-Dimethoxybenzyl)oxy)methyl)-1H-1,2,3-triazol-1-yl)ethyl)amino)methyl)-5,8a-dimethyl-3,3a,6,7,8,8a,9,9a-octahydronaphtho [2,3-b]furan-2(5H)-one (**7d**).

Yellow oil (83%). ^1^H NMR (400 MHz, CDCl_3_) δ 7.67 (1H, s, H-18), 6.89 (1H, s, H-23), 6.87 (1H, d, *J* = 8.1 Hz, H-27), 6.80 (1H, d, *J* = 8.0 Hz, H-26), 5.05 (1H, d, *J* = 2.9 Hz, H-7), 4.70–4.68 (1H, m, H-9), 4.62 (2H, s, H_2_-21), 4.50 (2H, s, H_2_-20), 4.44–4.42 (2H, m, H_2_-17), 3.85, 3.83 (6H, both s, H_3_-28, H_3_-29), 3.18–3.15 (1H, m, H-8), 3.09–3.02 (2H, m, H_2_-16), 2.96–2.89 (2H, m, H_2_-15), 2.74–2.70 (1H, m, H-11), 2.44–2.41 (1H, m, H-3), 2.05 (1H, dd, *J* = 14.8 Hz, *J* = 3.1 Hz, H-10a), 1.82–1.74 (1H, m, H-2a), 1.56–1.37 (6H, m, H-1, H-2b, H-6, H-10b), 1.18 (3H, s, H_3_-13), 1.07 (3H, d, *J* = 7.6 Hz, H_3_-14). ^13^C NMR (100 MHz, CDCl_3_) δ 177.63 (C-12), 150.88 (C-4), 148.69 (C-25), 148.37 (C-24), 144.69 (C-19), 130.04 (C-22), 123.11 (C-18), 120.31 (C-27), 114.55 (C-7), 111.03 (C-26), 110.62 (C-23), 77.16 (C-9), 72.13 (C-21), 63.01 (C-20), 55.61 (C-29), 55.57 (C-28), 49.86 (C-17), 48.89 (C-15), 45.90 (C-16), 45.36 (C-11), 42.34 (C-10), 41.85 (C-6), 38.13 (C-3), 37.31 (C-8), 32.70 (C-5), 32.50 (C-2), 28.34 (C-13), 22.68 (C-14), 16.51 (C-1). IR (KBr) *v*_max_ 2929, 1758 (C=O), 1593, 1517, 1265, 1157, 1028, 810, 756 cm^−1^. Mass spectrum (LC-MS ESI), *m*/*z* (*I*_rel_, %): 525.50 (100) [M + H]^+^. Anal. Calc. for C_29_H_40_N_4_O_5_·CH_2_Cl_2_: C, 59.11; H, 6.94; N, 9.19%. Found: C, 59.45; H, 6.74; N, 9.18%.

### 3.5. Animals

The animals were kept under standard conditions in accordance with Directive 2010/63 EU of the European Parliament and of the Council of the European Union of September 22, 2010, on the protection of animals used for scientific purposes. All experiments with animals were carried out in compliance with international principles and norms in accordance with the decisions of the Commission on Biological Ethics of the IPAC RAS (protocol No. 63 dated 10 October 2022).

### 3.6. Isolation of Rat Liver Mitochondria

Rat liver mitochondria were isolated by standard differential centrifugation [51] using buffer A for isolation and buffer B for washing.

Buffer A composition (pH = 7.4): 225 mM mannitol, 75 mM sucrose, 10 mM HEPES, 0.5 mM EGTA, 0.5 mM EDTA.

Buffer B composition (pH = 7.4): 225 mM mannitol, 75 mM sucrose, 10 mM HEPES, 20 mM EGTA.

The yield of mitochondria ranged from 80 to 100 mg protein/mL buffer in the liver sediment. The functional activity of rat liver mitochondria remained constant for 4 h. The concentration of mitochondrial protein was determined by Biuret standard method [52]. In the assay we used mitochondria at a final concentration of 0.5 mg protein/mL.

### 3.7. Determination of Mitochondrial Membrane Potential

The transmembrane potential (∆Ψm) of rat liver mitochondria was estimated using safranine-A fluorescence at 485/590 nm (ex/em), using a Victor 3 multi-plate analyzer (Perkin Elmer, Waltham, MA, USA) [53]. Mitochondria (0.5 mg protein/1 mL buffer) were incubated in the buffer (pH = 7.4) containing 225 mM mannitol, 75 mM sucrose, 10 mM HEPES, 20 mM EGTA, and 1 mM KH_2_PO_4_. As a respiratory substrate of complex II, 5 mM potassium succinate was used in the presence of an inhibitor of complex I, 1 mM rotenone. Against the organelle energization background, 100 mM of the studied compounds were added in the 1% solution in DMSO form. Addition of 25 mM calcium chloride (CaCl_2_) was used to dissipate the ∆Ψm.

### 3.8. Cell Culture

In this study we used human cell culture of tumor origin—SH-SY5Y (neuroblastoma), HeLa (cervical tumor), Hep-2 (larynx carcinoma), A549 (adenocarcinomic human alveolar basal epithelial cells); and normal cell line Hek 293 (human embryonic kidneys)), provided by the Laboratory of Tumor Cell Genetics of the Scientific Research Institute of Carcinogenesis, N.N. Blokhin National Medical Research Center of Oncology, as well as the Institute of Cytology of the Russian Academy of Sciences. While being grown for experiments, cells were cultured at 37 °C in a humidified CO_2_ atmosphere (5%) in a nutrient medium DMEM (PanEco, Moscow, Russia) and MEM (PanEco, Moscow, Russia), containing fetal bovine serum (10% by volume) (Thermo Fisher Scientific, Paisley, UK), Glutamax (2 mM) (Gibco, Renfrewshire, UK), and penicillin-streptomycin (1% by volume) (PanEco, Moscow, Russia).

### 3.9. Determination of Cell Viability

Cell viability was determined by MTT test as described earlier [54]. Cells were seeded in a 96-well plate in the amount of 1 × 10^4^ cells/200 µL and cultured at 37 °C in a humidified CO_2_ atmosphere (5%) in a nutrient medium DMEM (PanEco, Moscow, Russia) and MEM (PanEco, Moscow, Russia). After 24 h of incubation, 2 mL aliquots of test compounds (0.1 to 100 mM) dissolved in DMSO were added to the cell cultures, and cells were cultured under the same conditions for 24 h. The final content of DMSO in the well did not exceed 1% and did not have a toxic effect on the cells. DMSO also was added to the control wells in a volume of 1%.

After 24 h, 20 mL of MTT (3-(4,5-dimethylthiazol-2-yl)-2,5-diphenyltetrazolium bromide, 5 mg/mL) was added to each well and the plates were additionally incubated for 2 h.

Using a plate analyzer (Cytation3, BioTek Instruments Inc., Winooski, VT, USA), the optical density was determined at 530 nm. The concentration value causing 50% inhibition of cell population growth (IC_50_) was determined from dose-dependent curves.

### 3.10. Glycolysis Flux Assay

The ability of compounds to suppress anaerobic glycolysis was studied using the Agilent Seahorse XF96e Analyzer (Seahorse Bioscience, Billerica, MA, USA) by the level of hydrogen proton production in the studied samples on cell lines of neuroblastoma SH-SY5Y [55].

The 30,000 SH-SY5Y cells per well were plated in an XF cell culture microplate (Seahorse Bioscience, Billerica, MA, USA). After plating cells, the microplate was kept overnight in a 37 °C, 5% CO_2_ incubator. On the following day the medium was aspirated, the cells washed and placed in serum-free, pyruvate-free and glucose-free medium (pH = 7.4). The microplate was kept for 45 min again in a 37° C, 5% CO_2_ incubator.

When SH-SY5Y cells were analyzed, we followed the procedure described in the Seahorse Glycolysis Stress Test kit. Briefly, initial extracellular acidification rate (ECAR) measurements were taken in the absence of glucose. Next, compounds were added from port A to a concentration of 100 mM in accordance with the experimental scheme, from port B glucose was added to each well to a final concentration of 10 mM. This was followed by an injection of oligomycin from port C so that the final concentration of oligomycin in each well was 1 mM. Lastly, a 25 mM final concentration of 2-deoxyglucose was injected from port D.

Data for each experimental condition derive from values obtained from a minimum of 5 separate wells.

### 3.11. Evaluation of Synthesized Compounds Pharmacokinetic Parameters

Pharmacokinetic parameters (ADME/Tox) were determined using the ‘QikProp’ subroutine [56]. The methodology for predicting molecular properties is based on the Monte Carlo method. The molecules were compared with the properties of known drugs: the prediction was based on the generation of a ‘similarity matrix’ of fragments of the studied molecule.

A full description and interpretation of the pharmacokinetic parameters that were calculated for the synthesized compounds is presented below:

#star—the smaller (the maximum value is 5), the more this compound looks like a potential drug molecule: drug-like;

QPlogPo/w is the predicted value of the octanol/water partial coefficient: the value should be in the range from −2.0 to 6.5;

QPlogS is the predicted solubility value, logS, where the S—value in mol/dm^3^ should be in the range from −6.5 to 0.5;

QPPCaco—evaluation of permeability through cells of the Caco-2 line (continuous line of heterogeneous cells of human epithelial colorectal adenocarcinoma) in nm/s. Caco-2 cells are a model of the intestinal and blood barrier. Prediction of the permeability of inactive transport. A value of less than 25 indicates poor permeability, more than 500 indicates good;

QPlogBB—assessment of permeability through the blood–brain barrier (predicted brain/blood distribution coefficient for oral administration). The value should be in the range from −3.0 to 1.2;

Percent Human Oral Absorption is a predicted oral absorption based on a quantitative model of multiple linear regression. A value below 20% is considered a bad indicator, above 80%—a good one;

Rule Of Five—the number of violations of the Lipinski rule of five, in which the value of MW should be less than 500, QPlogPo/w—less than 5, the number of donors and acceptors of hydrogen bonds should not exceed 5 and 10, respectively. It is believed that compounds satisfying this rule are similar to drugs;

Rule Of Three—the number of violations of Jorgensen’s rule of three, in which the values of the solubility logarithm should be greater than −5.7, the permeability through the Caco-2 cell line is more than 22 nm/s, and the number of primary metabolites is less than 7. Compounds without violations of this rule are more likely to be biologically active when administered orally.

## 4. Conclusions

Using the methodology of “click” chemistry, a convenient approach has been developed for the synthesis of conjugates based on dehydrocostuslactone and alantolactone with aromatic polyalkoxy derivatives. In order to search for potential antitumor agents among the synthesized compounds, their effect on the survival of cells of both tumor and normal origin was studied and some mechanisms of their cytotoxic action were established. It was found that the length of the 1,2,3-triazole spacer, as well as the nature of the pharmacophore parts of the conjugates, affect the severity of the toxic effect in relation to cell cultures. The elongation of the spacer in compounds based on alantolactone **7a**–**d** leads to an increase in cytotoxic properties without a selective effect on tumor cells. In turn, among the substances containing dehydrocostuslactone, the most toxic were compounds with a short spacer **10a**–**d**, which selectively and most effectively suppressed the survival of cells of tumor origin. Such a cytotoxic effect of the obtained conjugates may be due to their ability to exert a modulating effect on mitochondria, triggering processes associated with cell death through the mitochondria-dependent apoptosis pathway, as well as suppression of the glycolysis process, the main way of obtaining energy by tumor cells. Interestingly, the analysis of the possible mechanisms of the toxic action of these compounds also revealed a clear correlation with the results of the study of the cytotoxic profile. Thus, the most pronounced ability to cause the dissipation of the transmembrane potential of mitochondria and inhibit the parameters of the glycolytic function of transformed cells was noted for the conjugates of alantolactone with a long spacer and dehydrocostuslactone with the opposite spacer length. Moreover, additional studies of the pharmacokinetic parameters (ADME/Tox) of synthesized compounds made it possible to position all substances as potential drugs that meet the “drug-like” criteria.

The obtained results confirm the assumption that variation in the length of the 1,2,3-triazole spacer, as well as the nature of the pharmacophore moieties used in the development of conjugates based on sesquiterpene lactones and polyalkoxybenzenes, can be considered as a promising strategy for the search for potential antitumor agents with a pronounced cytotoxic effect, associated with the ability to depolarize the mitochondrial membrane and inhibit glycolysis.

## Figures and Tables

**Table 1:** Cytotoxic potential of dehydrocostuslactone and alantolactone conjugates with alkoxy-substituted benzyl azides.

Compounds	IC_50_, µM				
	SH-SY5Y	HeLa	Hep-2	A549	Hek 293
**6a**	37.35 ± 0.28	62.40 ± 0.05	88.12 ± 2.66	85.65 ± 0.38	56.98 ± 1.24
**6b**	36.32 ± 2.38	61.52 ± 0.12	>100	>100	56.42 ± 1.03
**6c**	94.90 ± 1.08	82.4 ± 1.14	>100	>100	59.75 ± 1.37
**6d**	57.77 ± 1.71	>100	>100	>100	69.47 ± 0.54
**7a**	33.59 ± 1.27	38.96 ± 1.27	55.68 ± 0.52	29.34 ± 1.37	38.87 ± 1.23
**7b**	41.74 ± 1.73	55.14 ± 0.94	67.16 ± 0.14	47.33 ± 1.52	42.31 ± 1.09
**7c**	39.98 ± 1.32	46.55 ± 0.81	62.56 ± 0.72	53.11 ± 0.25	46.25 ± 0.22
**7d**	45.17 ± 1.24	81.01 ± 3.17	91.05 ± 2.85	83.52 ± 1.09	35.39 ± 0.29
**10a**	19.31 ± 0.57	18.02 ± 0.32	>100	>100	58.04 ± 0.07
**10b**	16.14 ± 0.46	16.60 ± 0.59	26.28 ± 0.38	25.20 ± 3.44	56.65 ± 0.05
**10c**	89.47 ± 2.57	79.59 ± 0.33	>100	>100	66.29 ± 0.08
**10d**	21.83 ± 0.02	26.40 ± 2.39	82.16 ± 1.15	>100	>100
**11a**	71.78 ± 2.00	88.86 ± 0.21	>100	95.93 ± 1.50	61.43 ± 0.10
**11b**	60.38 ± 0.39	72.79 ± 0.07	>100	70.33 ± 1.17	49.55 ± 0.33
**11c**	>100	88.22 ± 1.27	>100	>100	67.91 ± 0.65
**11d**	56.08 ± 1.06	89.35 ± 0.40	>100	91.22 ± 1.54	59.58 ± 1.84
Arglabin	34.02 ± 2.48	20.00 ± 0.00 [35]	29.92 ± 0.93	-	78.00 ± 2.05

**Table 2:** Calculated pharmacokinetic (ADME/Tox) parameters of synthesized compounds.

Title	#Stars	QP logPo/w	QP logS	QPP Caco	QP logBB	% Human Oral Absorption	Rule of Five	Rule of Three
**6a**	1	3.969	−3.8	366.597	−0.465	83.12	1	1
**6b**	1	4.625	−5.66	187.542	−1.027	94.711	0	1
**6c**	2	4.754	−5.889	189.541	−1.117	82.592	1	2
**6d**	1	4.719	−5.574	257.607	−0.919	84.771	1	1
**7a**	0	4.19	−5.204	297.515	−0.711	82.789	1	1
**7b**	0	4.287	−4.964	199.479	−0.932	93.208	0	1
**7c**	1	4.512	−5.48	182.961	−1.103	80.897	1	1
**7d**	2	4.649	−5.46	391.813	−0.696	87.616	1	1
**10a**	0	3.3	−2.088	405.689	−0.058	92.952	0	1
**10b**	1	4.397	−5.221	221.865	−0.651	94.684	0	1
**10c**	0	3.743	−4.252	281.813	−0.411	92.714	0	0
**10d**	1	4.408	−5.071	200.677	−0.745	93.963	0	1
**11a**	1	4.309	−5.32	193.287	−0.736	93.093	0	1
**11b**	0	4.338	−5.255	221.334	−0.736	94.317	0	1
**11c**	0	4.258	−4.825	241.762	−0.645	94.534	0	1
**11d**	1	4.613	−5.401	318.736	−0.561	100	0	1
Range of values	Less is better	(−2 ÷ 6.5)	(−6.5 ÷ 0.5)	>500	(−3 ÷ 1.2)	>80—large <20—small	<4	<3

## Data Availability

Not applicable.

## Supplementary Materials

The following supporting information can be downloaded at: https://www.mdpi.com/article/10.3390/molecules27238411/s1. NMR spectra of compound **3** S2; NMR spectra of compound **4**; NMR spectra of compound **5c** S4; NMR spectra of compounds **6a**–**d** S5–S8; NMR spectra of compounds **7a**–**d** S9–S12.

## Sample Availability

Samples of the compounds are available from the authors.
