# Supplementary material for: Design of Conjugates Based on Sesquiterpene Lactones with Polyalkoxybenzenes by “Click” Chemistry to Create Potential Anticancer Agents
Source: Molecules. 2022 Dec 1;27(23):8411. doi: 10.3390/molecules27238411 (PMC9738796; doi:10.3390/molecules27238411)
Supplement: Supplementary file 1 [file molecules-27-08411-s001.zip › molecules-2059728-supplementary.pdf]

molecules

Supplementary Materials

Design of Conjugates Based on Sesquiterpene Lactones with Polyalkoxybenzenes by "Click" Chemistry to Create Potential Anticancer Agents

Margarita E. Neganova [1,2], Ekaterina V. Smirnova [1], Elena V. Sharova [1], Oleg I. Artyushin [1], Yulia R. Aleksandrova [2], Ekaterina Yu. Yandulova [2], Natalia S. Nikolaeva [2] and Valery K. Brel [1,*]

[1] Nesmeyanov Institute of Organoelement Compounds, Russian Academy of Sciences, 119991 Moscow, Russia

[2] Institute of Physiologically Active Compounds at Federal Research Center of Problems of Chemical Physics and Medicinal Chemistry, Russian Academy of Sciences, 142432 Chernogolovka, Russia

* Correspondence: v_brel@mail.ru; Tel.: +7-(499)1356373

List of content:

1. NMR spectra of compound **3** S2

2. NMR spectra of compound **4** S3

3. NMR spectra of compound **5c** S4

4. NMR spectra of compounds **6a-d** S5-S8

5. NMR spectra of compounds **7a-d** S9-S12

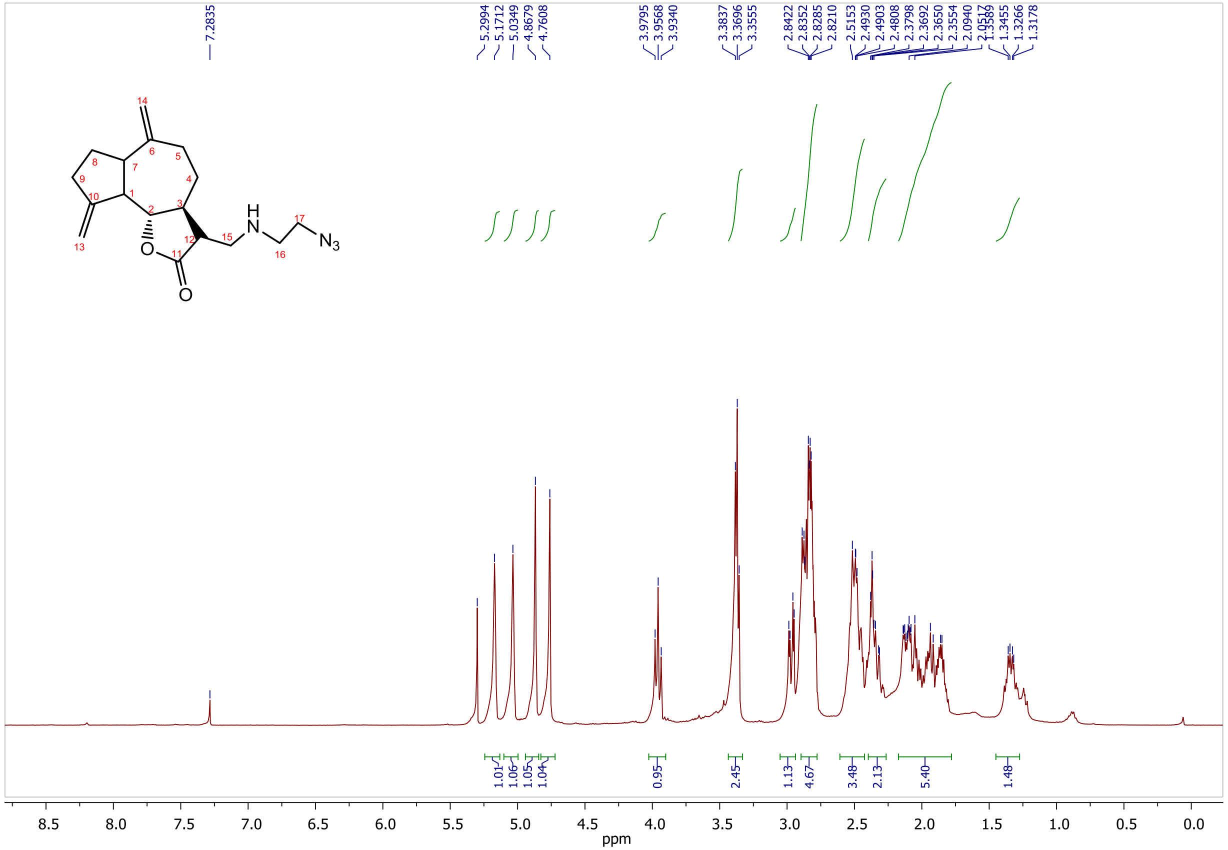

7.2835
5.2994
5.1712
5.0349
4.8679
4.7608
3.9795
3.9568
3.9340
3.3837
3.3696
3.3555
2.8422
2.8352
2.8285
2.8210
2.5153
2.4930
2.4903
2.4808
2.3798
2.3692
2.3650
2.3554
2.0940
2.0517
1.3589
1.3455
1.3266
1.3178
14
6
5
8
7
4
9
10
1
2
3
H
N
17
15
12
N3
13
O
11
16
O
1.01
1.06
1.05
1.04
0.95
2.45
1.13
4.67
3.48
2.13
5.40
1.48
8.5
8.0
7.5
7.0
6.5
6.0
5.5
5.0
4.5
4.0
3.5
3.0
2.5
2.0
1.5
1.0
0.5
0.0
ppm

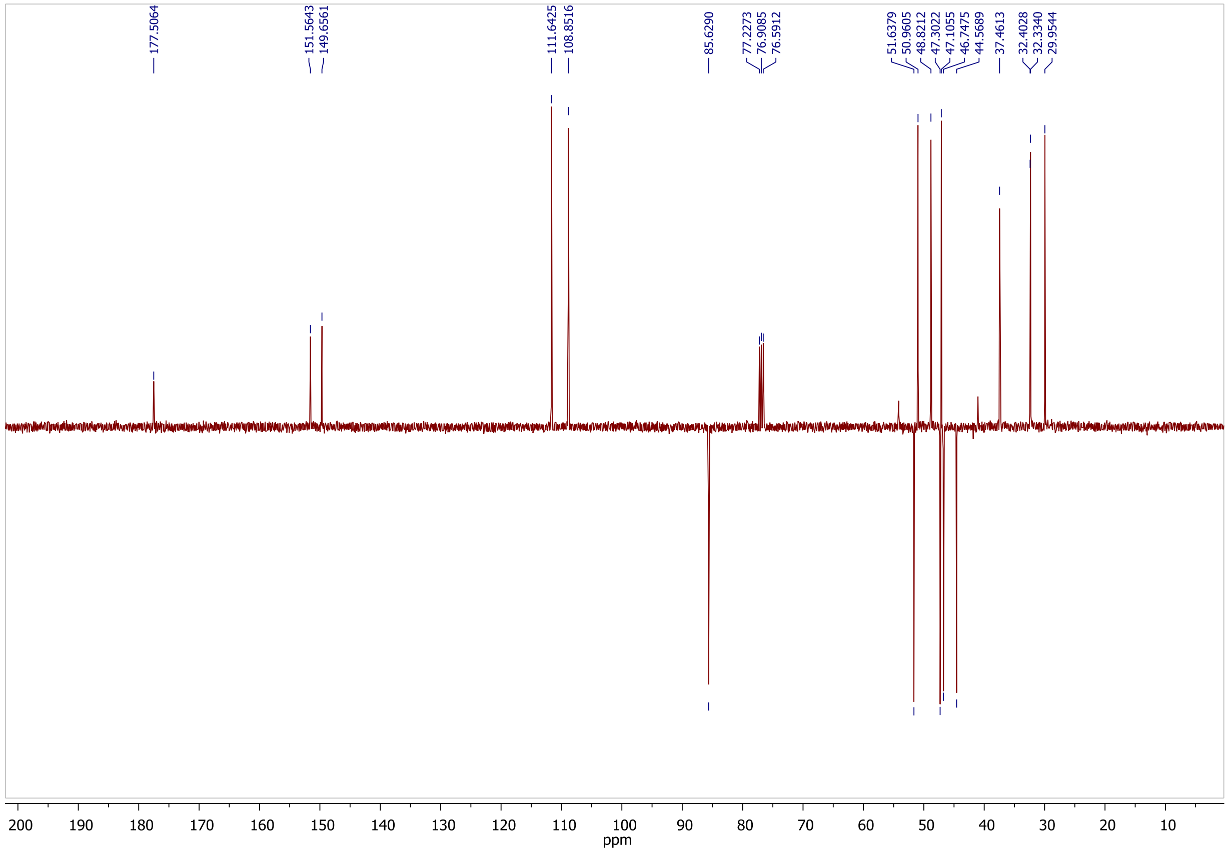

^{1}H and ^{13}C NMR spectra of compound **3** ($CDCl_3$)

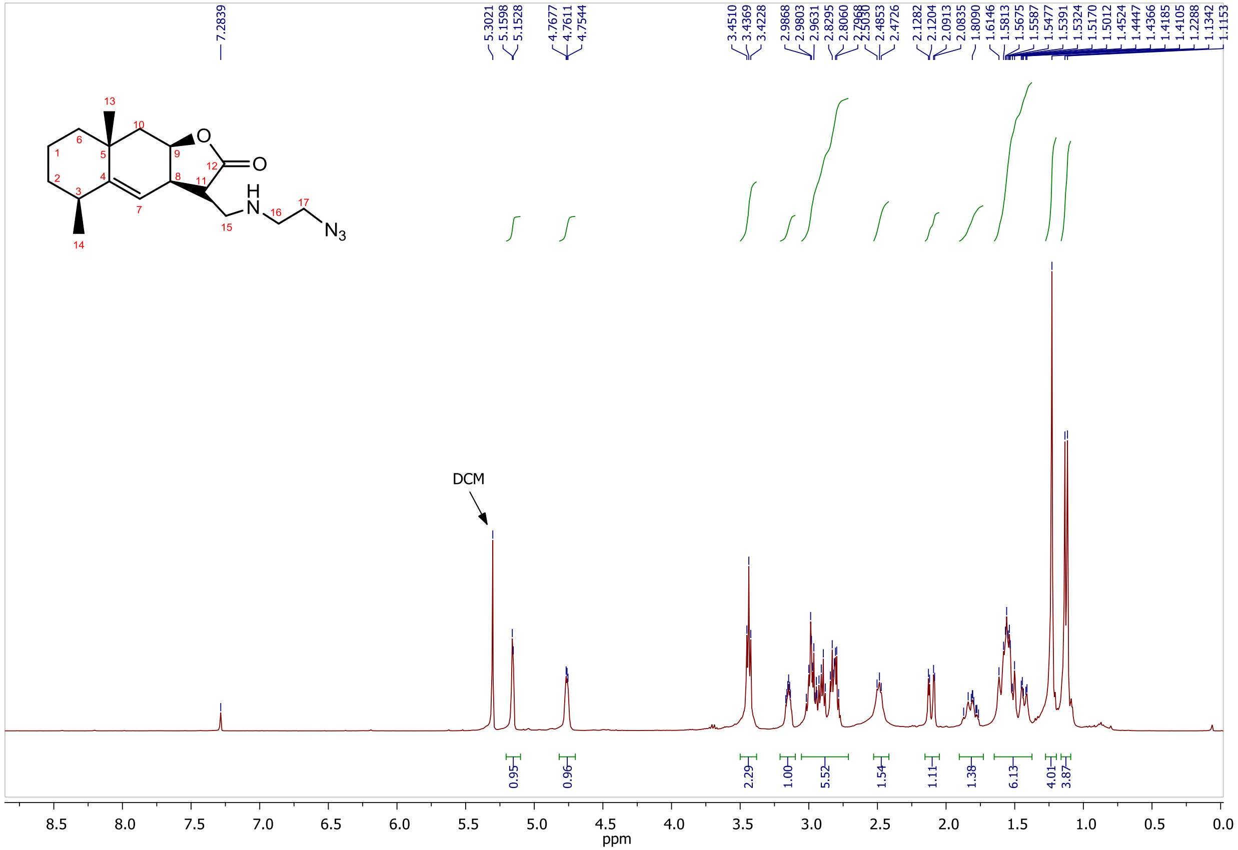

7.2839
5.3021
5.1598
5.1528
4.7677
4.7611
4.7544
3.4510
3.4369
3.4228
2.9868
2.9803
2.9631
2.8295
2.8060
2.4853
2.4726
2.1282
2.1204
2.0913
2.0835
1.8090
1.6146
1.5813
1.5675
1.5587
1.5477
1.5391
1.5324
1.5170
1.5012
1.4524
1.4447
1.4366
1.4185
1.4105
1.2288
1.1342
1.1153
O
O
H
N
N3
1
2
3
4
5
6
7
8
9
10
11
12
13
14
15
16
17
DCM
0.95
0.96
2.29
1.00
5.52
1.54
1.11
1.38
6.13
4.01
3.87
8.5 8.0 7.5 7.0 6.5 6.0 5.5 5.0 4.5 4.0 3.5 3.0 2.5 2.0 1.5 1.0 0.5 0.0
ppm

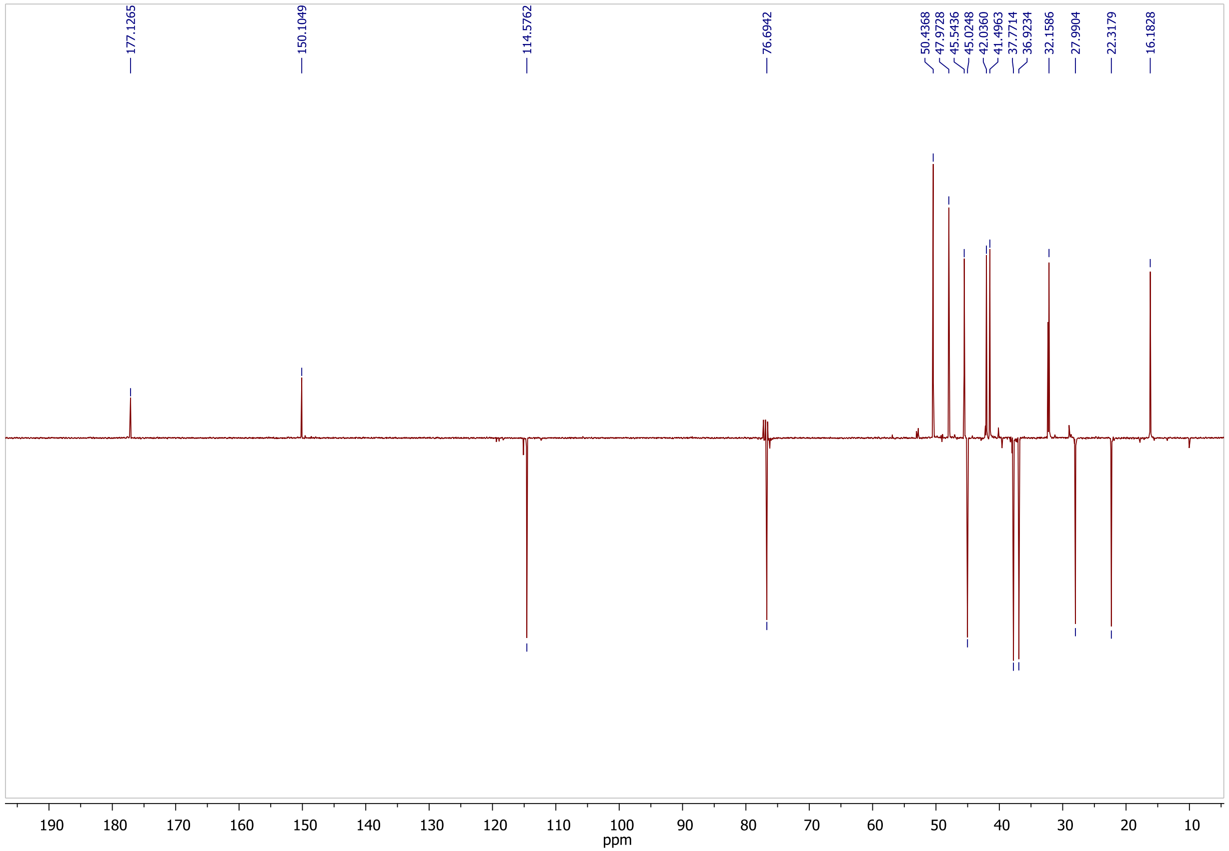

^{1}H and ^{13}C NMR spectra of compound **4** ($CDCl_3$)

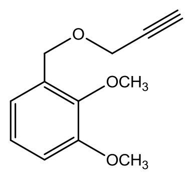

O
OCH3
OCH3

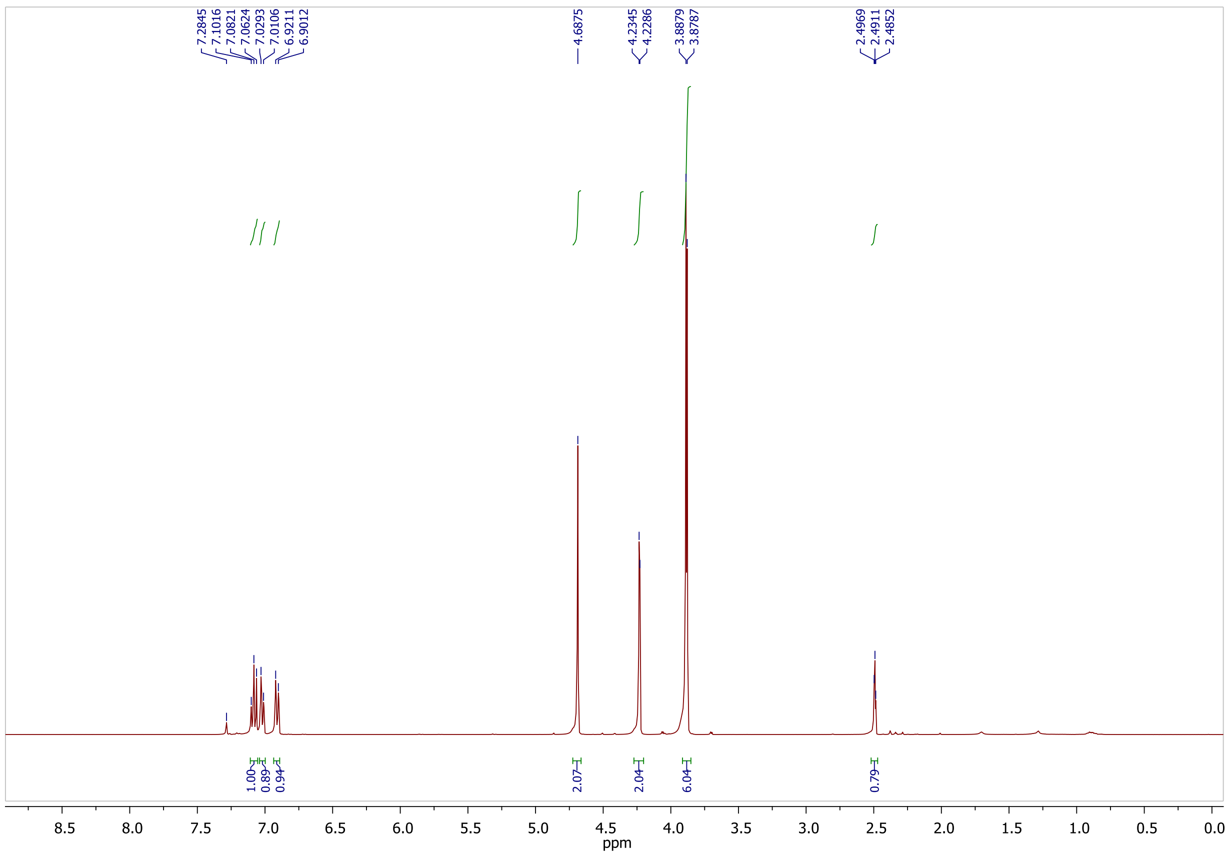

7.2845
7.1016
7.0821
7.0624
7.0293
7.0106
6.9211
6.9012
4.6875
4.2345
4.2286
3.8879
3.8787
2.4969
2.4911
2.4852
1.00
0.89
0.94
2.07
2.04
6.04
0.79
8.5
8.0
7.5
7.0
6.5
6.0
5.5
5.0
4.5
4.0
3.5
3.0
2.5
2.0
1.5
1.0
0.5
0.0
ppm

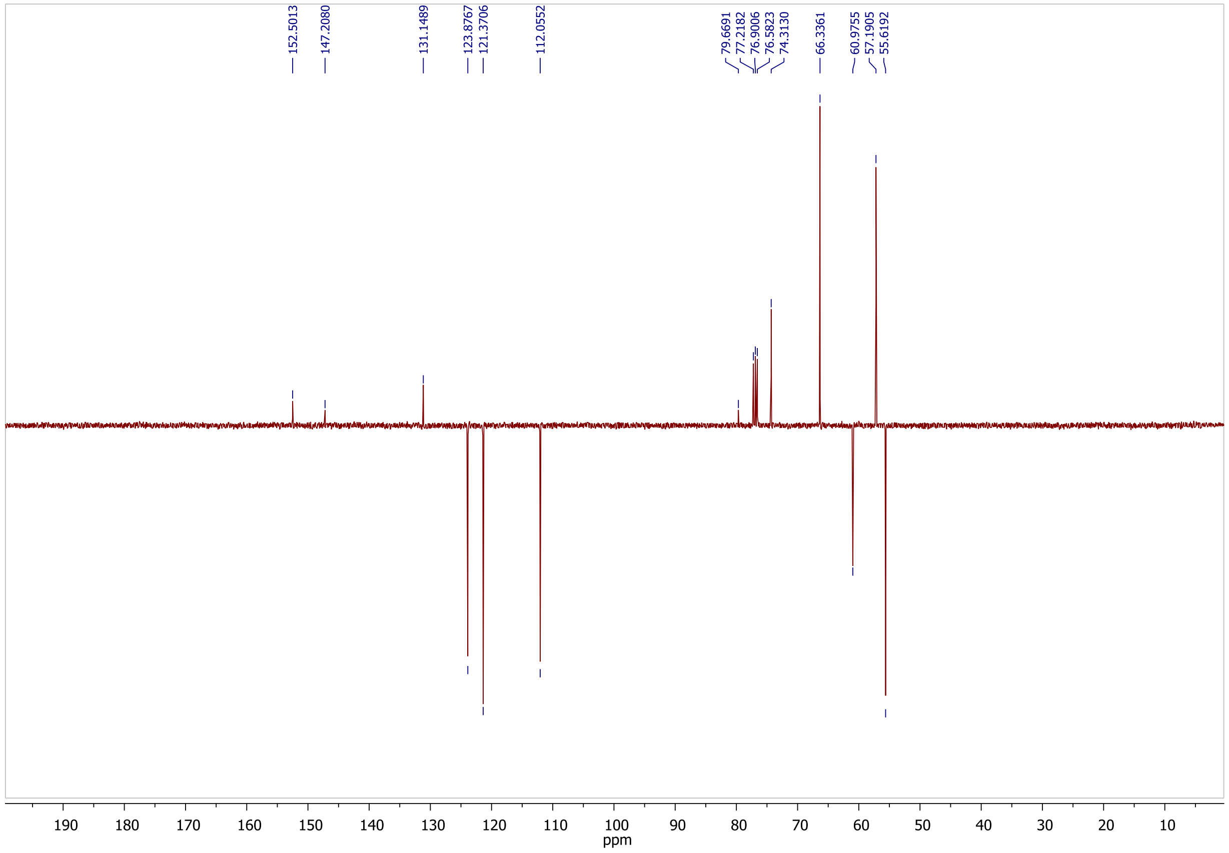

^{1}H and ^{13}C NMR spectra of compound **5c** ($CDCl_3$)

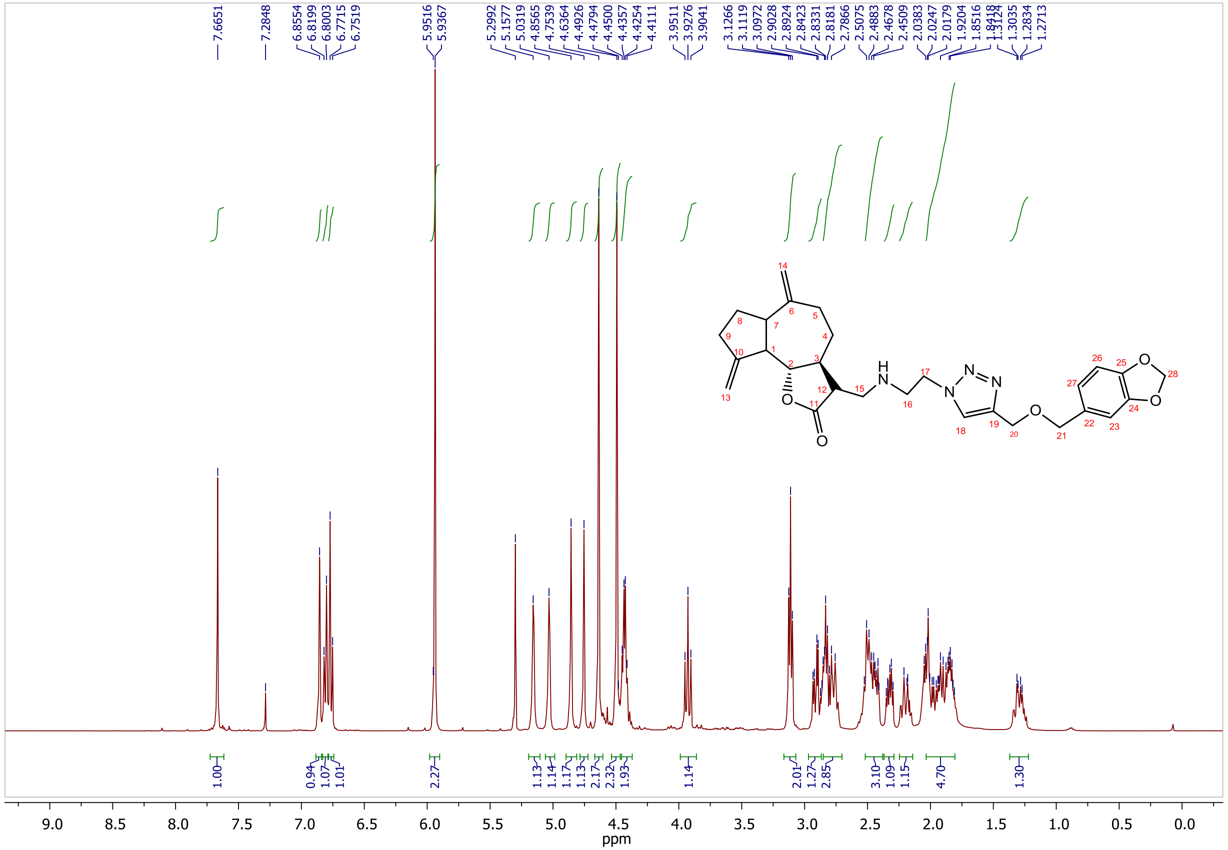

7.6651
7.2848
6.8554
6.8199
6.8003
6.7715
6.7519
5.9516
5.9367
5.2992
5.1577
5.0319
4.8565
4.7539
4.6364
4.4926
4.4794
4.4500
4.4357
4.4254
4.4111
3.9511
3.9276
3.9041
3.1266
3.1119
3.0972
2.9028
2.8924
2.8423
2.8331
2.8181
2.7866
2.5075
2.4883
2.4678
2.4509
2.0383
2.0247
2.0179
1.9204
1.8516
1.3124
1.3035
1.2834
1.2713
1.00
0.94
1.07
1.01
2.27
1.13
1.14
1.17
1.13
2.17
2.32
1.93
1.14
2.01
1.27
2.85
3.10
1.09
1.15
4.70
1.30
9.0
8.5
8.0
7.5
7.0
6.5
6.0
5.5
5.0
4.5
4.0
3.5
3.0
2.5
2.0
1.5
1.0
0.5
0.0
ppm

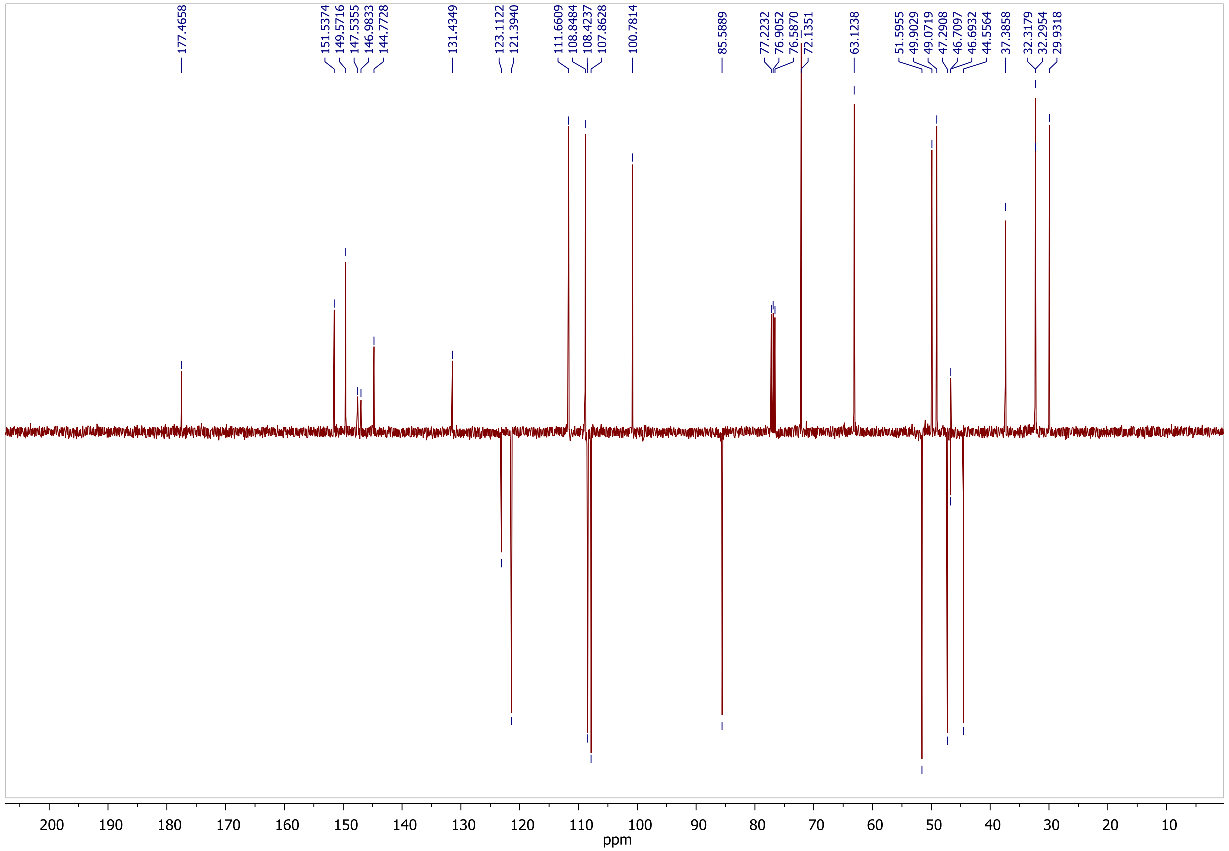

^1H and ^{13}C NMR spectra of compound **6a** ($CDCl_3$)

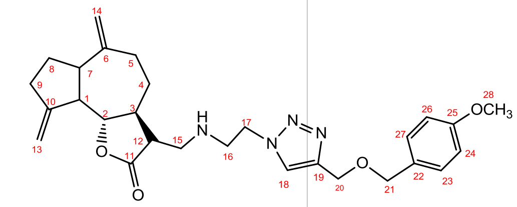
14
8
6
5
7
9
4
10
1
3
2
12
15
H
N
17
N
N
N
16
13
O
11
O
18
19
20
O
21
22
23
24
25
26
27
28
OCH3

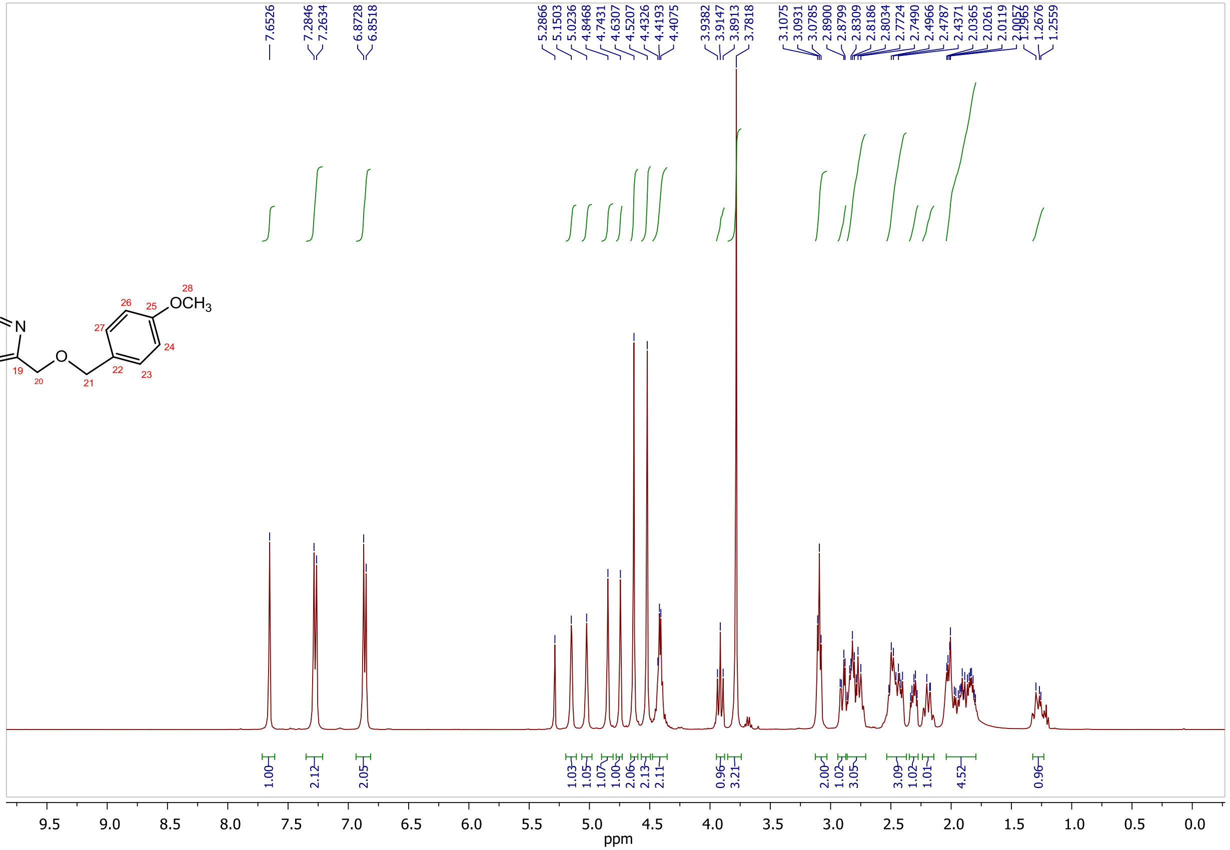
7.6526
7.2846
7.2634
6.8728
6.8518
5.2866
5.1503
5.0236
4.8468
4.7431
4.6307
4.5207
4.4326
4.4193
4.4075
3.9382
3.9147
3.8913
3.7818
3.1075
3.0931
3.0785
2.8900
2.8799
2.8309
2.8186
2.8034
2.7724
2.7490
2.4966
2.4787
2.4371
2.0365
2.0261
2.0119
1.2676
1.2559
1.00
2.12
2.05
1.03
1.05
1.07
1.00
2.06
2.13
2.11
0.96
3.21
2.00
1.02
3.05
3.09
1.02
1.01
4.52
0.96
9.5
9.0
8.5
8.0
7.5
7.0
6.5
6.0
5.5
5.0
4.5
4.0
3.5
3.0
2.5
2.0
1.5
1.0
0.5
0.0
ppm

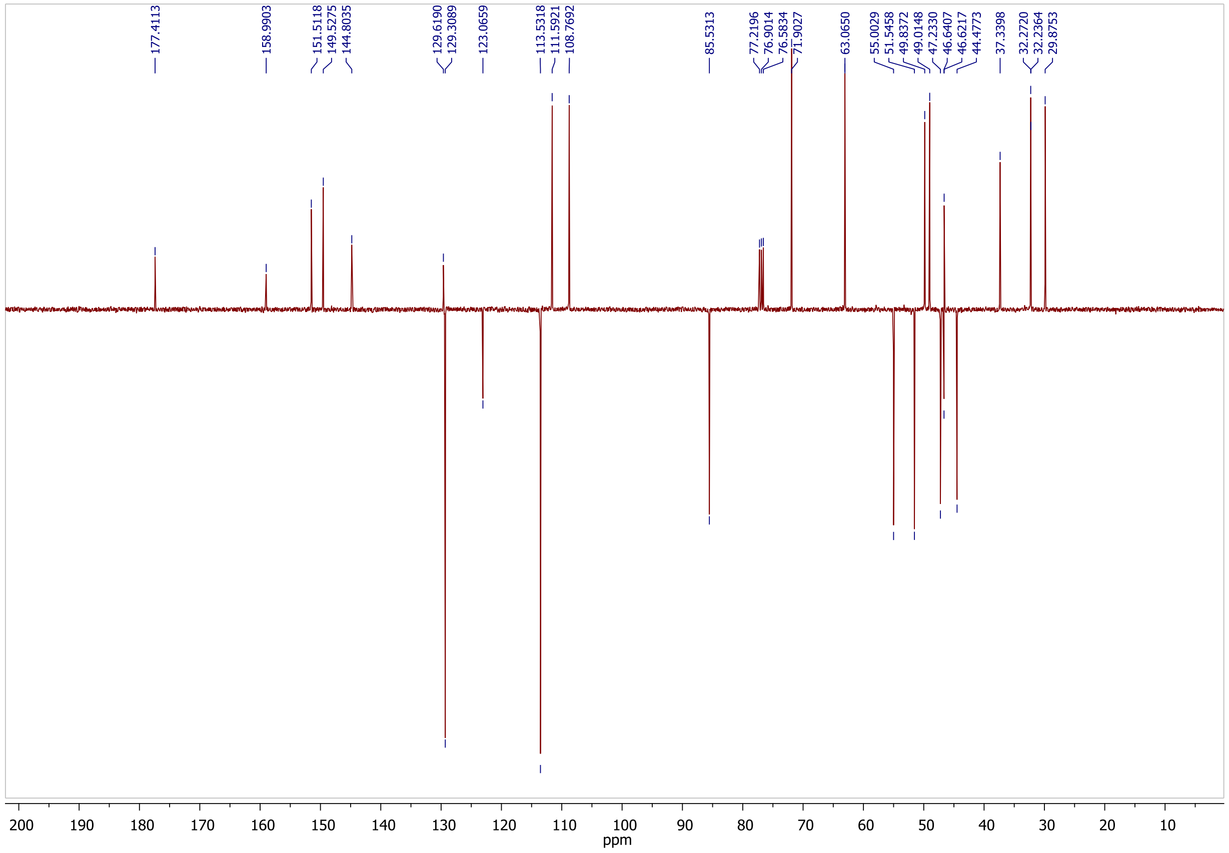

^{1}H and ^{13}C NMR spectra of compound **6b** ($CDCl_3$)

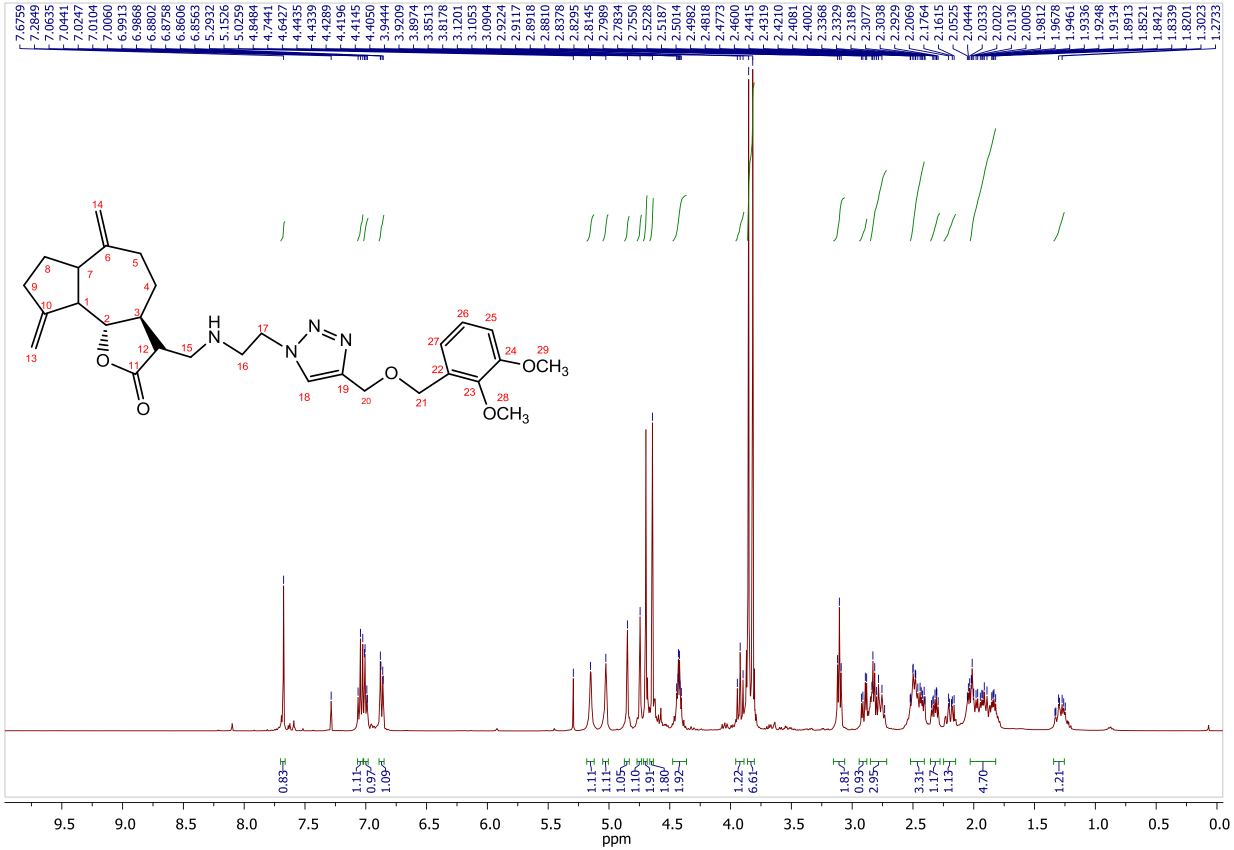

7.6759 7.2849 7.0635 7.0441 7.0247 7.0104 7.0060 6.9913 6.9868 6.8802 6.8758 6.8606 6.8563 5.2932 5.1526 5.0259 4.8484 4.7441 4.6427 4.4435 4.4339 4.4289 4.4196 4.4145 4.4050 3.9444 3.9209 3.8974 3.8513 3.8178 3.1201 3.1053 3.0904 2.9224 2.9117 2.8918 2.8810 2.8378 2.8295 2.8145 2.7989 2.7834 2.7550 2.5228 2.5187 2.5014 2.4982 2.4818 2.4773 2.4600 2.4415 2.4319 2.4210 2.4081 2.4002 2.3368 2.3329 2.3189 2.3077 2.3038 2.2929 2.2069 2.1764 2.1615 2.0525 2.0444 2.0333 2.0202 2.0130 2.0005 1.9812 1.9678 1.9461 1.9336 1.9248 1.9134 1.8913 1.8521 1.8421 1.8339 1.8201 1.3023 1.2733
0.83 1.11 0.97 1.09 1.11 1.11 1.05 1.10 1.91 1.80 1.92 1.22 6.61 1.81 0.93 2.95 3.31 1.17 1.13 4.70 1.21
9.5 9.0 8.5 8.0 7.5 7.0 6.5 6.0 5.5 5.0 4.5 4.0 3.5 3.0 2.5 2.0 1.5 1.0 0.5 0.0
ppm

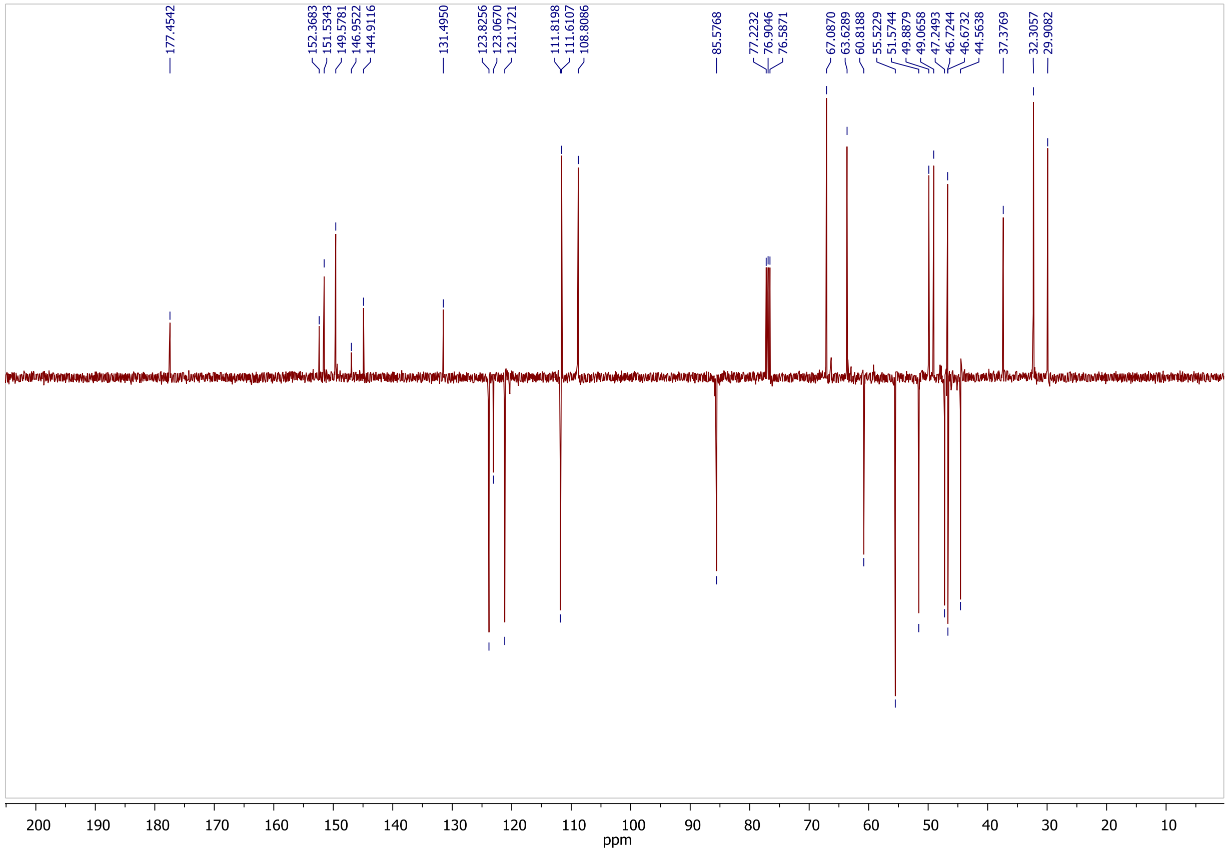

^{1}H and ^{13}C NMR spectra of compound **6c** ($CDCl_3$)

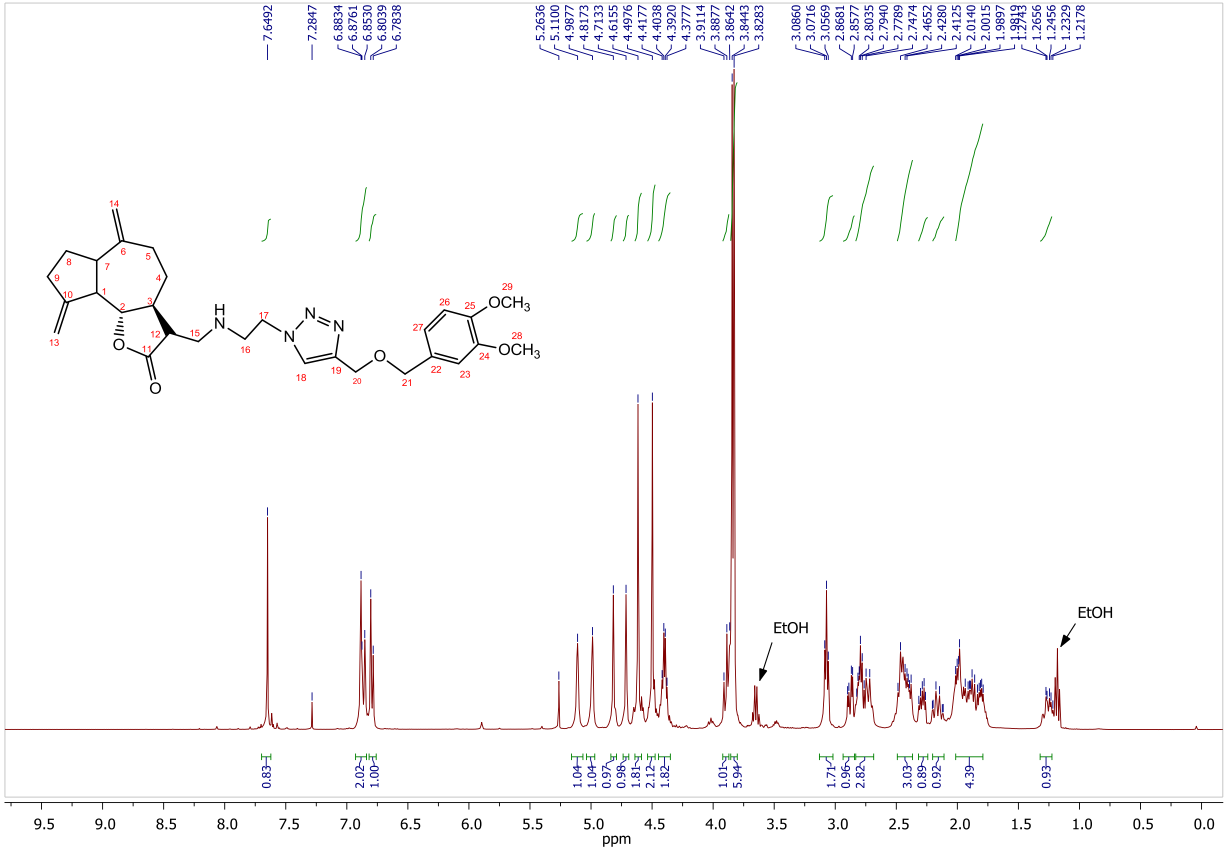

7.6492
7.2847
6.8834
6.8761
6.8530
6.8039
6.7838
5.2636
5.1100
4.9877
4.8173
4.7133
4.6155
4.4976
4.4177
4.4038
4.3920
4.3777
3.9114
3.8877
3.8642
3.8443
3.8283
3.0860
3.0716
3.0569
2.8681
2.8577
2.8035
2.7940
2.7789
2.7474
2.4652
2.4280
2.4125
2.0140
2.0015
1.9897
1.2656
1.2456
1.2329
1.2178
EtOH
EtOH
0.83
2.02
1.00
1.04
1.04
0.97
0.98
1.81
2.12
1.82
1.01
5.94
1.71
0.96
2.82
3.03
0.89
0.92
4.39
0.93
9.5
9.0
8.5
8.0
7.5
7.0
6.5
6.0
5.5
5.0
4.5
4.0
3.5
3.0
2.5
2.0
1.5
1.0
0.5
0.0
ppm
OCH3
OCH3
O
O
O
H
N
N
N
N

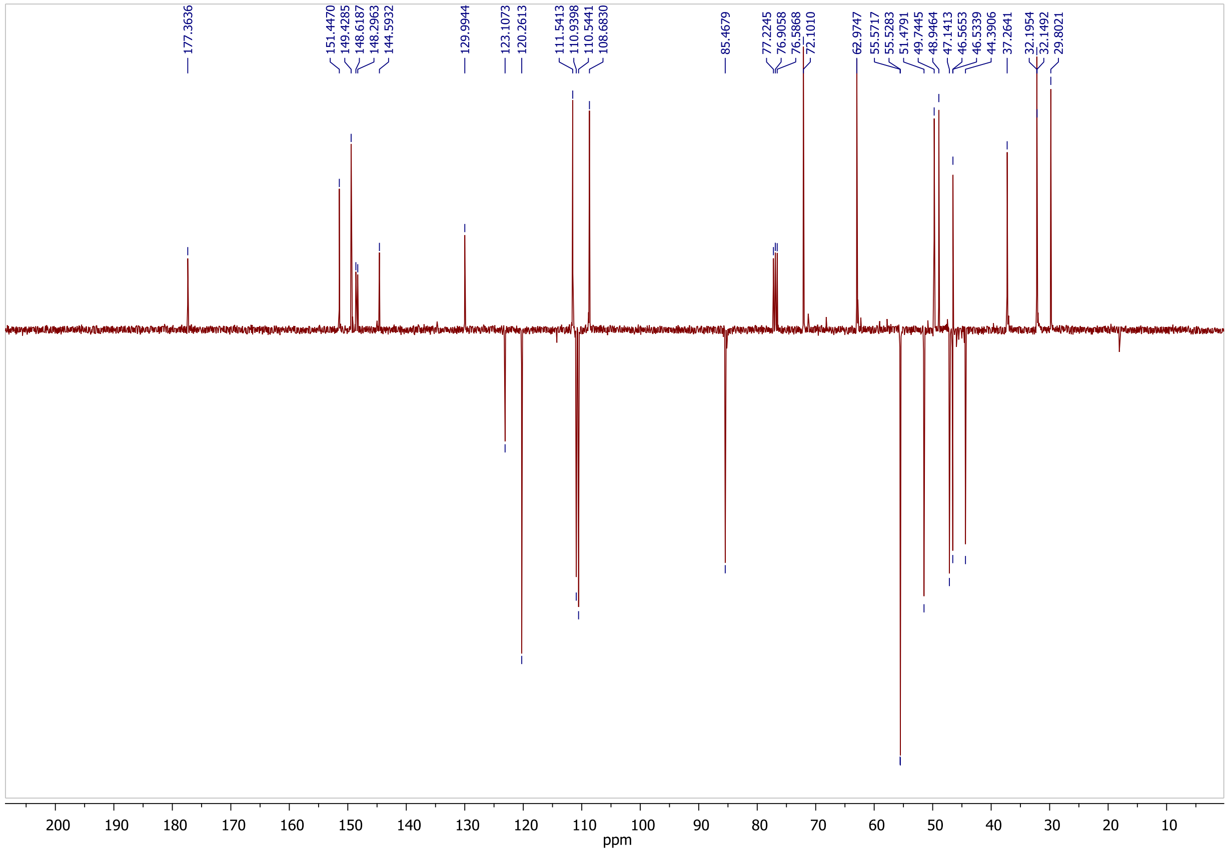

^{1}H and ^{13}C NMR spectra of compound **6d** ($CDCl_3$)

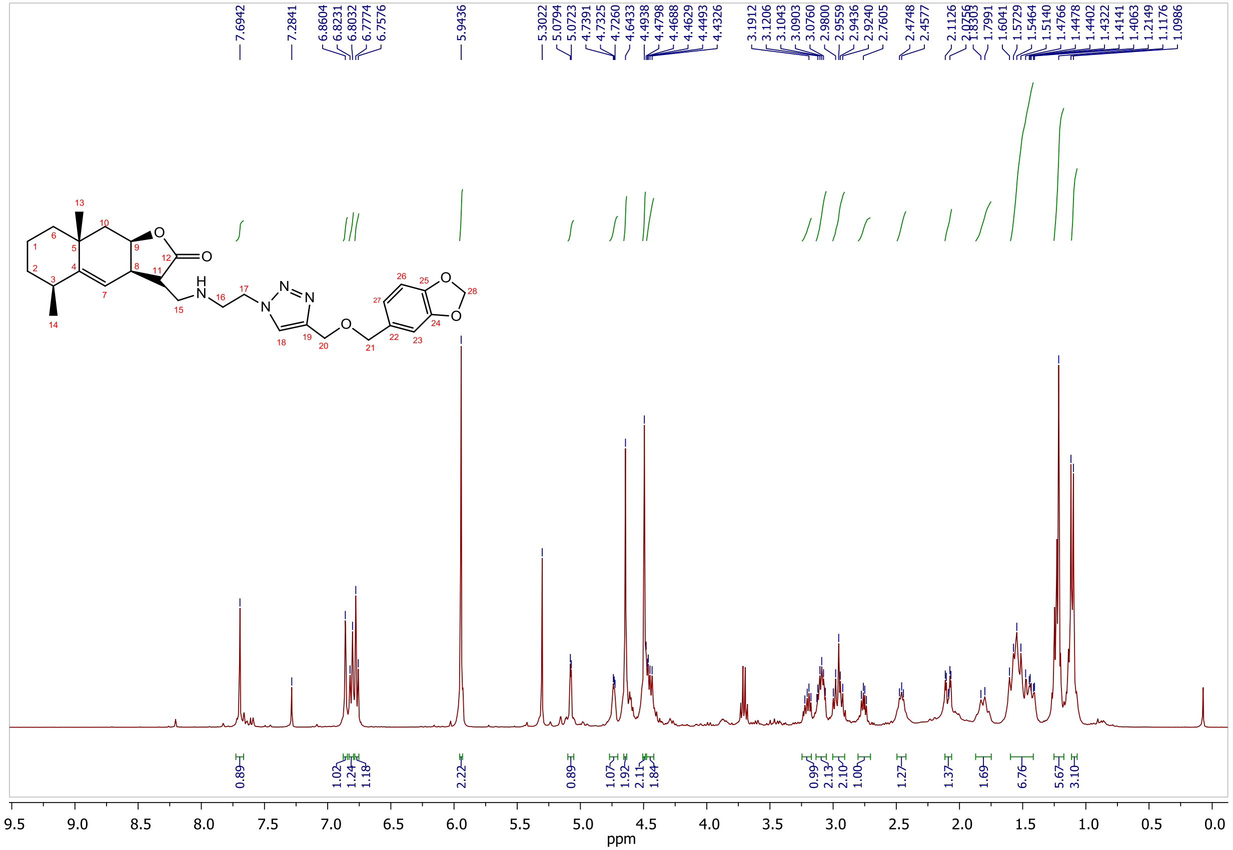

7.6942
7.2841
6.8604
6.8231
6.8032
6.7774
6.7576
5.9436
5.3022
5.0794
5.0723
4.7391
4.7325
4.7260
4.6433
4.4938
4.4798
4.4688
4.4629
4.4493
4.4326
3.1912
3.1206
3.1043
3.0903
3.0760
2.9800
2.9559
2.9436
2.9240
2.7605
2.4748
2.4577
2.1126
1.7991
1.6041
1.5729
1.5464
1.5140
1.4766
1.4478
1.4402
1.4322
1.4141
1.4063
1.2149
1.1176
1.0986
0.89
1.02
1.24
1.18
2.22
0.89
1.07
1.92
2.11
1.84
0.99
2.13
2.10
1.00
1.27
1.37
1.69
6.76
5.67
3.10
9.5 9.0 8.5 8.0 7.5 7.0 6.5 6.0 5.5 5.0 4.5 4.0 3.5 3.0 2.5 2.0 1.5 1.0 0.5 0.0
ppm

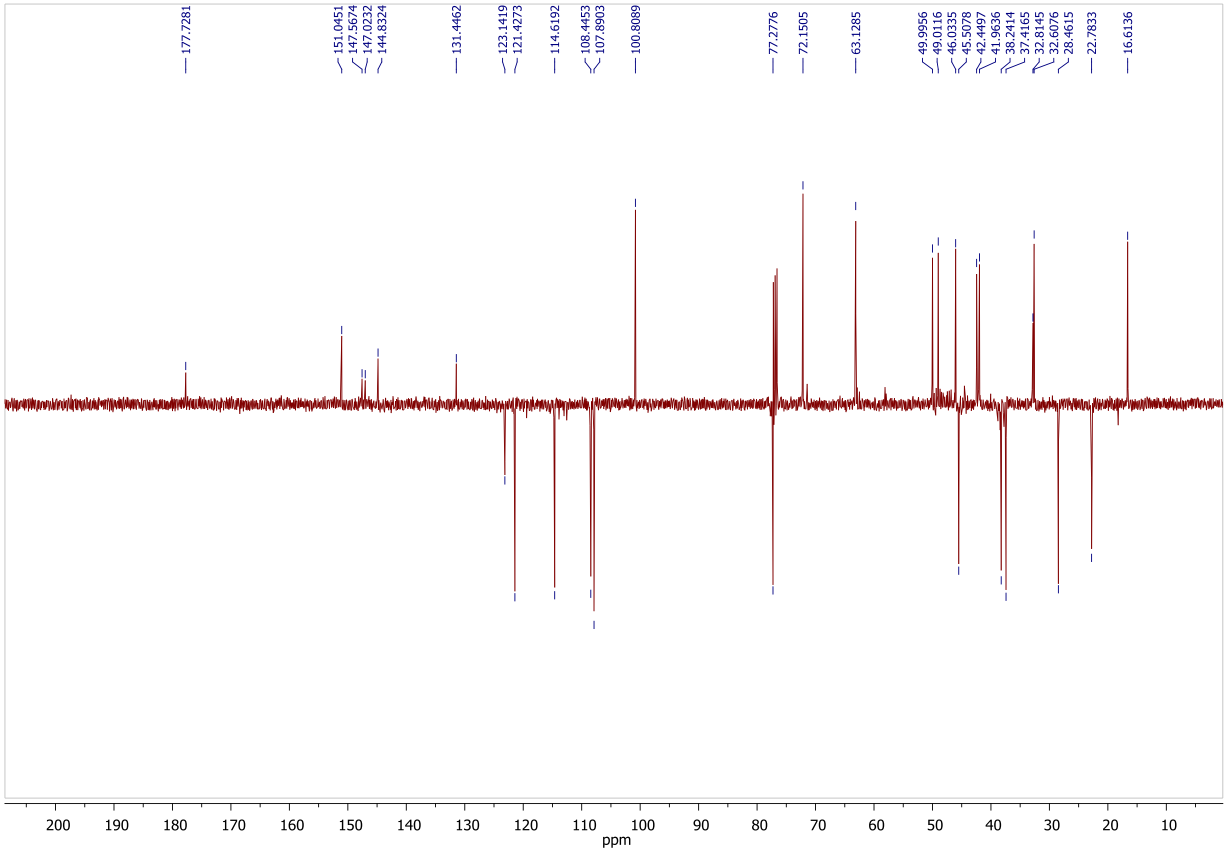

^{1}H and ^{13}C NMR spectra of compound **7a** ($CDCl_3$)

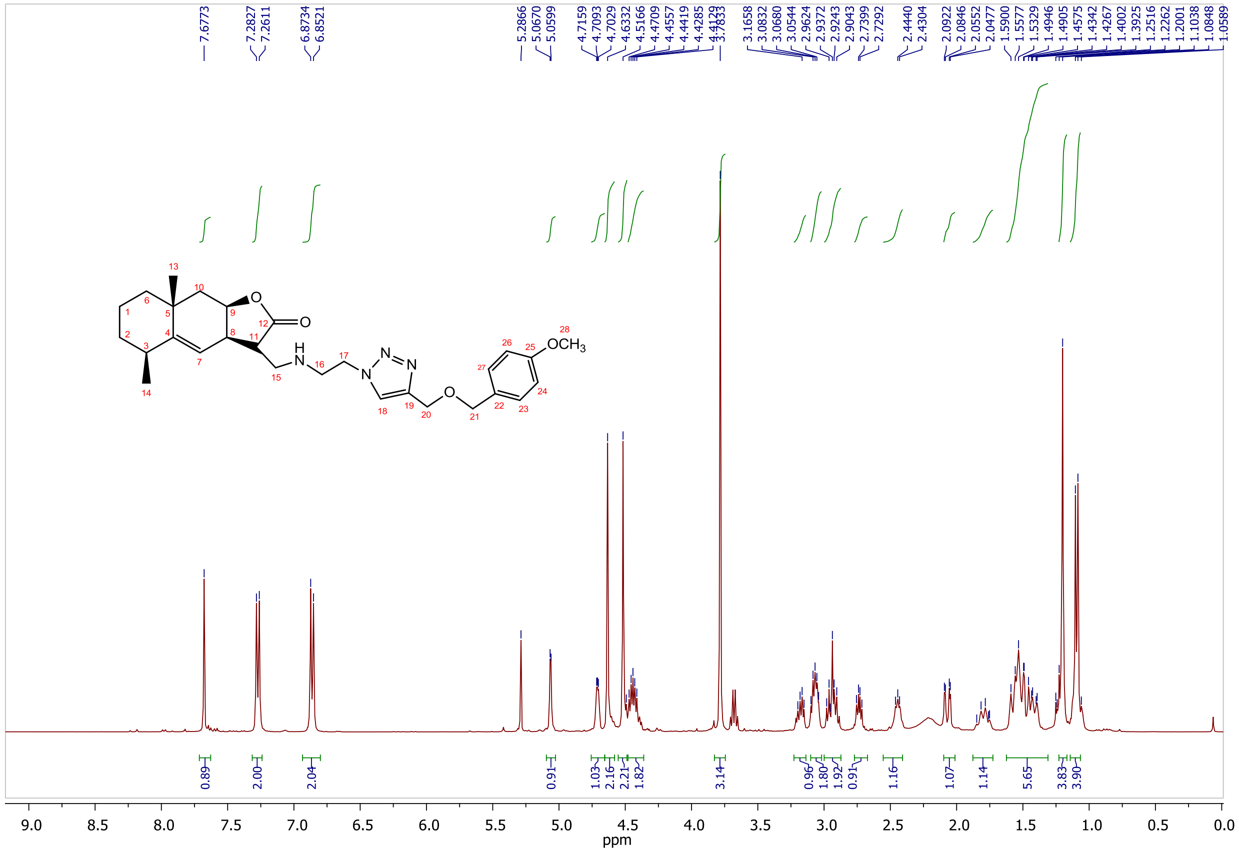

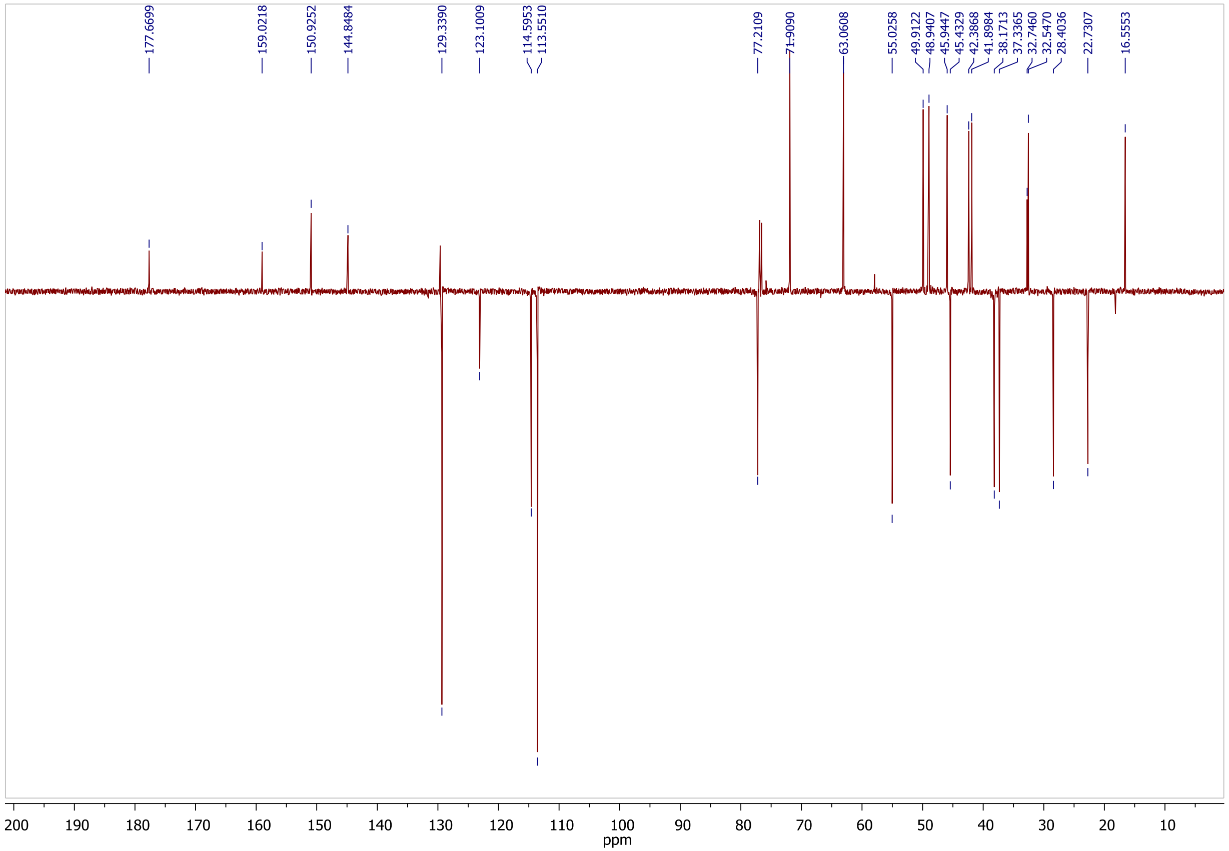

^{1}H and ^{13}C NMR spectra of compound **7b** ($CDCl_3$)

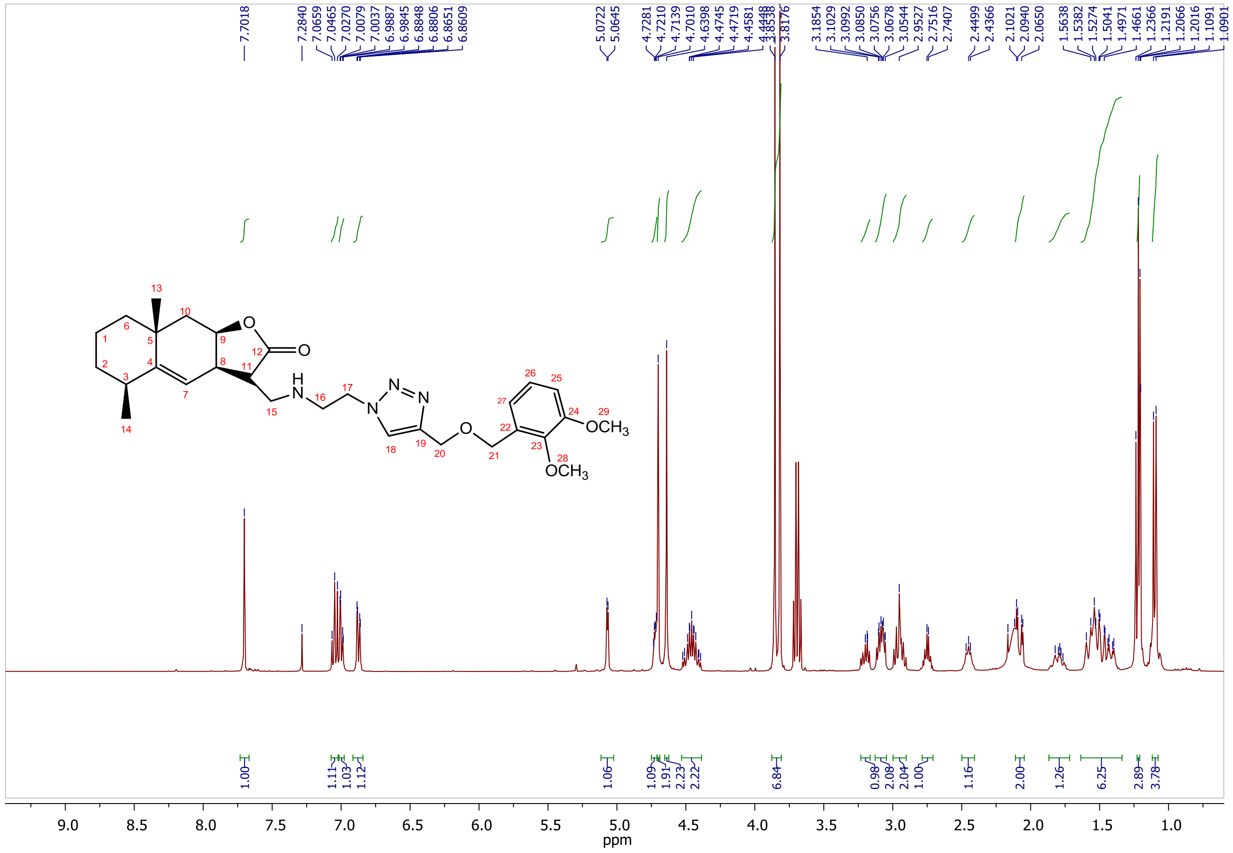

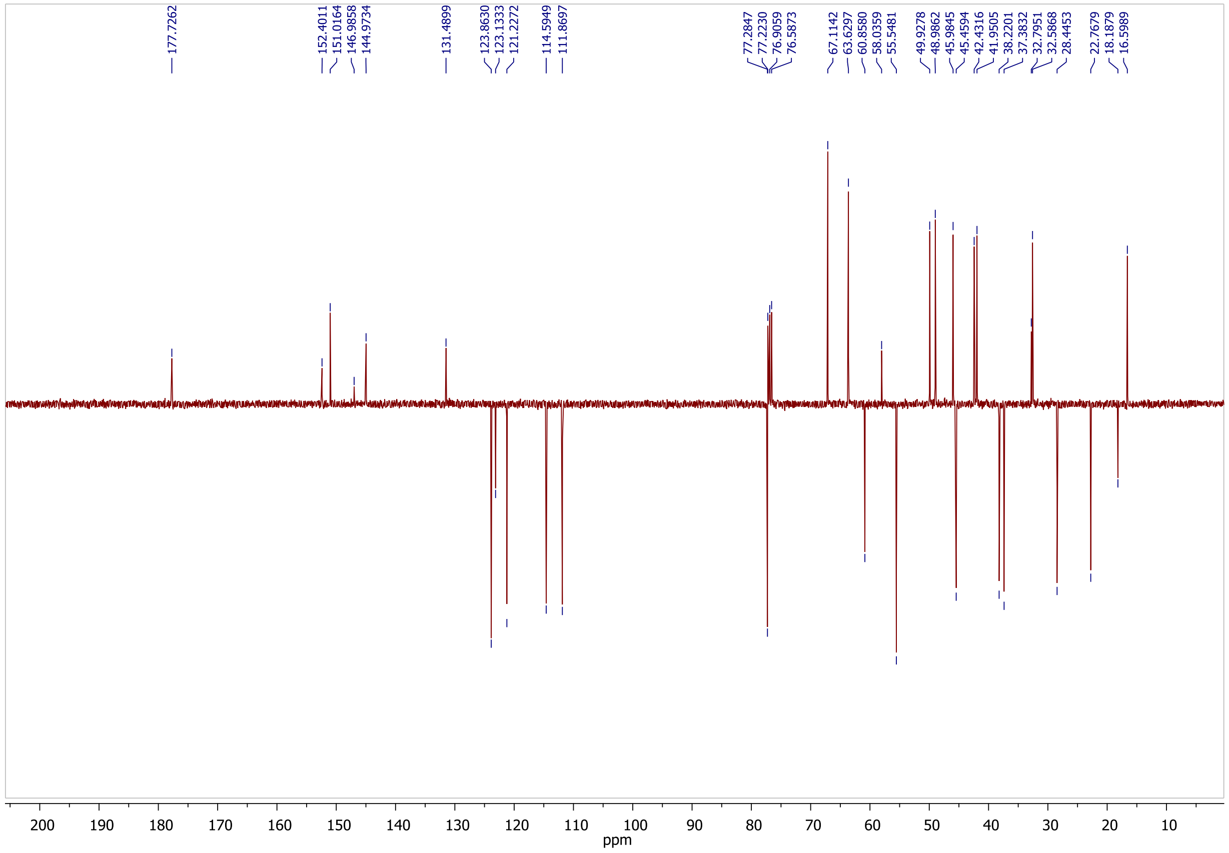

^{1}H and ^{13}C NMR spectra of compound **7c** ($CDCl_3$)

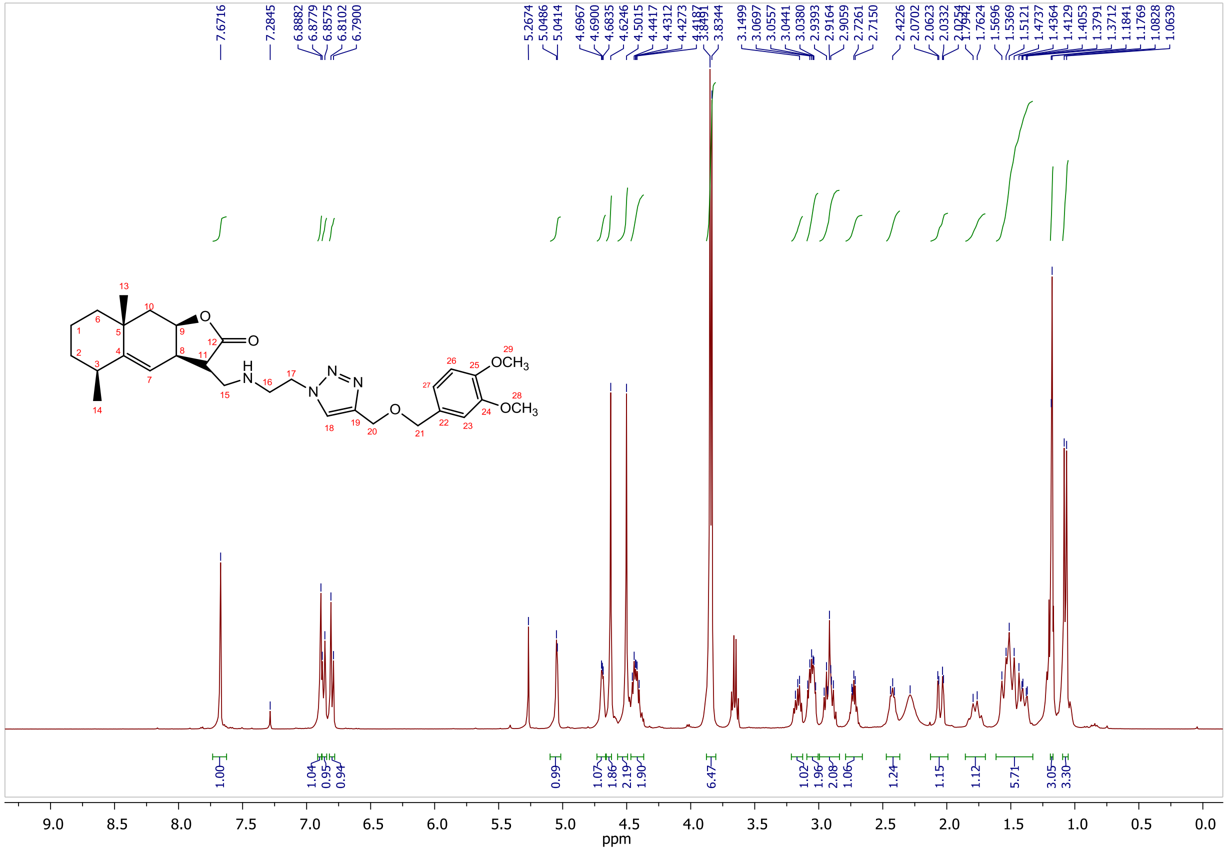
7.6716
7.2845
6.8882
6.8779
6.8575
6.8102
6.7900
5.2674
5.0486
5.0414
4.6967
4.6900
4.6835
4.6246
4.5015
4.4417
4.4312
4.4273
3.8344
3.1499
3.0697
3.0557
3.0441
3.0380
2.9393
2.9164
2.9059
2.7261
2.7150
2.4226
2.0702
2.0623
2.0332
1.7624
1.5696
1.5369
1.5121
1.4737
1.4364
1.4129
1.4053
1.3791
1.3712
1.1841
1.1769
1.0828
1.0639
O
OCH3
OCH3
N
1.00
1.04
0.95
0.94
0.99
1.07
1.86
2.19
1.90
6.47
1.02
1.96
2.08
1.06
1.24
1.15
1.12
5.71
3.05
3.30
9.0 8.5 8.0 7.5 7.0 6.5 6.0 5.5 5.0 4.5 4.0 3.5 3.0 2.5 2.0 1.5 1.0 0.5 0.0
ppm

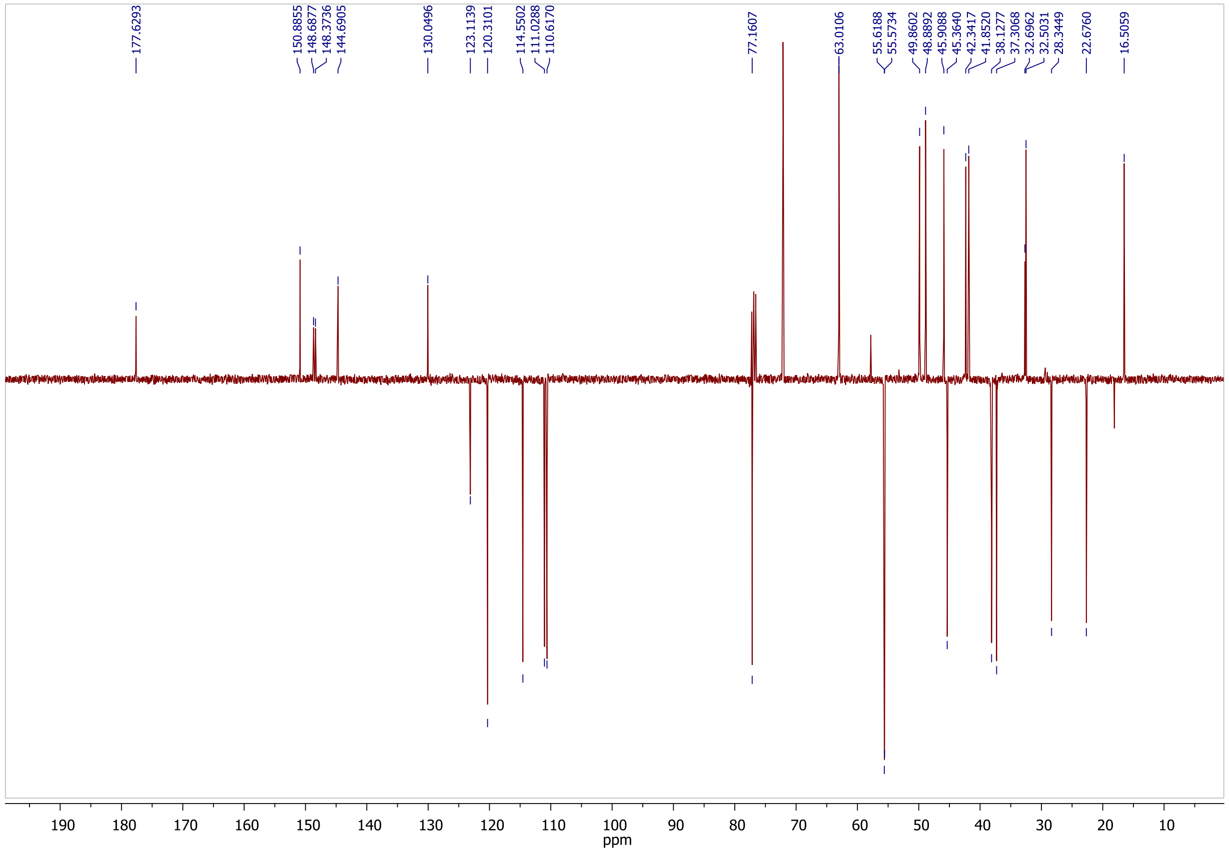

^{1}H and ^{13}C NMR spectra of compound **7d** ($CDCl_3$)
